# Effect of Seasonality on Microbiological Variability of Raw Cow Milk from Apulian Dairy Farms in Italy

**DOI:** 10.1128/spectrum.00514-22

**Published:** 2022-08-16

**Authors:** Giuseppe Celano, Maria Calasso, Giuseppe Costantino, Mirco Vacca, Arianna Ressa, Olga Nikoloudaki, Pasquale De Palo, Francesco Maria Calabrese, Marco Gobbetti, Maria De Angelis

**Affiliations:** a Department of Soil, Plant and Food Sciences, University of Bari A. Moro, Valenzano, Italy; b Department of Veterinary Medicine, University of Bari A. Moro, Valenzano, Italy; c Faculty of Science and Technology, Libera Università di Bolzano, Bolzano, Italy; University of Mississippi

**Keywords:** raw cow milk, 16S rRNA gene-based metataxonomic, seasonal microbiota, dairy farm management, microbiota metabolic predictions, metabolic predictions

## Abstract

Raw cow milk is one of the most complex and unpredictable food matrices shaped by the interaction between biotic and abiotic factors. Changes in dairy farming conditions impact the quality and safety of milk, which largely depend on seasonality. Changes in microbiome composition and relative metabolic pathways are derived from microbial interactions, as well as from seasonality, mammary, and extramammary conditions (e.g., farm management and outdoor environment). Breeding data from >600 Apulian farms were examined, and the associated physicochemical parameters were processed by a reductionist approach to obtain a raw cow milk sample subset. We investigated the microbiological variability in cultivable and 16S rRNA sequencing microbiota as affected by seasonal fluctuations at two time points (winter and summer seasons). We identified families (*Xanthomonadaceae*, *Enterobacteriaceae*, and *Pseudomonadaceae*) whose increased abundance during winter may cause a shift toward a pathobiont microbial niche that leads to lower milk quality. Apulian summer season conditions were advantageous to the presence of specific taxa, i.e., *Streptococcaceae* (i.e., *Lactococcus*) and *Limosilactobacillus fermentum*, which in turn may favor better milk preservation.

**IMPORTANCE** The strength of this study lies in the microbiological characterization of a wide range of farm management data to achieve a more comprehensive framework of Apulian milk. Specific regional pedoclimatic and management conditions impact the taxa present and their abundances within this ecological food niche. The obtained results lay the groundwork for comparison with other worldwide extensive farming areas.

## INTRODUCTION

Raw milk is a complex substrate enriched in four key components, specifically, water, fats, proteins, and lactose, along with a multitude of other minor constituents related to minerals and vitamins (1). This peculiar chemical composition is essential for the nutritional needs of both human and animal offspring (2).

At the same time, the macro- and micronutrient composition represents an optimal substrate for the growth of a wide consortium spectrum in which bacteria, yeasts, and molds coexist (1). This microbial community contributes both to animal physiology and health status and, by exploiting its protechnological features, impacts the quality and preservation of dairy products. In contrast, deterioration and spoilage processes are linked to the dysbiotic and pathological sphere with related implications for consumers.

Milk microbiota is the result of a cumulative effect of variables that can be distinguished overall in mammary and extramammary tissues (2, 3). The variables in mammary tissue depend on the cow udder and teat surface microbial community, whose dynamic composition affects the breast tissue health status (2, 4). In contrast, extramammary variables are related to dairy farm environmental factors, including air, dust, feces, water, feed, milking stables, biofilm presence on milking tools, and cross-contamination effectors carried by dairy operators (3, 5–7). In this light, milk microbiota play a key role in determining and maintaining dairy process quality and reproducibility (8). More specifically, the metabolism resulting from microbial activity impacts the safety, organoleptic, and nutritional qualities of dairy products (4).

Changes in milk microbial taxa ratios include enrichment in psychrophilic species (e.g., Pseudomonas, *Aeromonas*, *Listeria*, Staphylococcus, *Enterococcus*, and *Enterobacteriaceae*) as well as in spore-forming species (e.g., the *Clostridium* species C. butyricum, C. tyrobutyricum, C. bejierinckii, C. sporogenes, and C. bifermentans). These taxa with higher abundances have been linked to increased spoilage and also occurrence of defects in later milk processing, principally due to proteinase and lipase activities leading to gas-forming fermentations (9–15). On the other hand, the presence of protechnological bacteria, mainly lactic acid bacteria (LAB), improve biopreservation (e.g., via bacteriocin production) and thus impact cheese safety qualities (16–19).

In addition to these factors, temperature and environmental moisture are the main parameters shaping microbial ecosystems. In fact, fluctuations in taxa abundances must be carefully screened based on seasonality. Environmental factors can influence the breast tissue microbiota which, in turn, can directly affect the microbial community of raw milk. Specifically, among these drivers, the ratio between grazing and housing times is used to quantify animal exposure to different environmental niches enriched in distinct microorganism communities that may be delivered to raw milk.

A more in-depth characterization of milk focused on understanding whether microbial profiles and related metabolic pathways are subjected to seasonal changes that can drive the quality of dairy products. Notably, the present study is aimed at profiling bovine raw milk microbiota as they are affected by seasonality in the specific southern Italian region of Apulia, featured for its own pedoclimatic conditions. Although the overall Apulian milk production is not comparable with other more competitive Italian and worldwide distribution areas, the production of protected designation of origin and protected geographic identification cheeses is pivotal in sustaining the local economy, where raw milk is often implied for high-quality production. Management profiles were obtained for a total of 638 Apulian dairy farms and restricted to a reduced subset of them (*n* = 23). In summary, we inspected physicochemical properties and the cultivable microbiota composition of milk, together with 16S rRNA taxa profiles and the related metabolic pathway predictions.

## RESULTS

### Dairy farm management data.

Based on an administered questionnaire, 638 dairy farms from Apulia were profiled for farming management, animal feeding, hygiene and milking practices, milk storage, and collecting conditions prior to processing (see Table S1 in the supplemental material). The great majority (95%) of the interviewed dairy farms adopted conventional management, whereas the remaining 5% were certified as organic. The majority of included farms (51%) had cattle herds composed of more than 100 cows, whereas the remainder harbored between 50 and 100 (25%) or fewer than 50 units (24%). Overall, the principal breeding types were Holstein Friesian (45%), Brown Swiss (32%), and Simmenthal (10%). The remaining 13% of farms reported more than one breeding type. Outdoor housing was prevalent in more than half of the farms (61%), whereas a mixed approach of indoor and outdoor housing was found for 33%. Year-round indoor housing was adopted by 6% of the interviewed farms. Ninety-four percent of farms allowed the cows to roam freely during the day (see Table S1). All farms provided ventilation throughout the year. With respect to feeding of calves, colostrum was administered in 22% of farms, and the same percentage was fed milk substitutes during weaning. For feeding management after weaning, pasture feed accounted for 26% during the winter, while hay and grains accounted for 39% and 35%, respectively. During the summer, a reduced pasture time (18%) and a relative increase in hay and grain feeding (41%) were reported. Notably, 61% of farmers allowed cows to graze during the lactation phase. Pre- and postdipping udder cleaning was adopted daily by 92% of farms. Automatic and robotized milking systems were present in 95% and 5% of farms, respectively (see Table S1). The overall farm per-day average quantity was over 100 quintals (56%; i.e., 10,000 kg, as 1 quintal is equivalent to 100 kg), with fewer than 5,000 kg in 39% of cases. Approximately 5% of farms declared milk production ranging between 5,000 and 10,000 kg/day. Considering these Apulian breeding system features, we also investigated specific strategies for management and feeding used during the warm season to reduce the impact of heat stress (see Table S2). These practices are related to coping strategies for heat stress, including fan and cooling system activation, frequency of and changes in the daily total mixed ration, and modification of the daily pasture program.

### Compositional properties of milk and clustering.

A total of 638 raw cow milks were analyzed in terms of physicochemical composition, nutritional components, and dairy technological parameters (see Table S3). The main milk components, i.e., the lactose, proteins, and fat, represented 4.73% ± 0.10%, 3.5% ± 0.24%, and 3.93% ± 0.67%, respectively (means ± standard deviations [SD]). Moreover, mean pH was 6.55 ± 0.03 and somatic cell (SC) counts per milliliter were 261.76 ± 45.30 SC/mL. In terms of dairy technological traits, the consistency of coagulum (the A30, i.e., the amplitude [in millimeters] of curd firmness 30 min after enzyme addition), firming time (the K20, i.e., the time [in minutes] for the amplitude to increase from 1.5 to 20 mm), and coagulation time (R, i.e., the time [in minutes] between enzyme addition and attainment of the 1.5-mm amplitude) were 23.55 ± 6.63 mm, 8.55 ± 1.26 min, and 30.22 ± 3.36 min, respectively.

The overall annual trend of compositional results related to physicochemical and biological markers (pH, somatic cells score, and acetone, β-hydroxybutyric acid [BHBA], urea, and citric acid levels) and macronutrients (lactose, proteins, caseins, and fat and its constituents) combined with farm management data were inspected using boxplot distributions (see Fig. S1). The interquartile ranges showed that acetone, urea, and citric acid had greater variability. Concerning the macronutrient constituent class, fats and proteins together with trans fatty acids and caseins exhibited the highest variability. The per-season physicochemical parameters of Apulian dairy milk production are reported as median values (see Table S4).

The complete panel of physicochemical parameters related to the overall annual production were considered descriptive variables and were included in a multivariate analysis, i.e., a discriminant principal component analysis (DPCA). The output of the find.clusters function within the adegenet R package was the Bayesian information criterion (BIC) curve obtained without superimposing any *a priori* group that showed how the 638 milk samples (from as many farms) could be clustered into seven clouds (see Fig. S2). Specifically, after evaluating the DPCA plot, we selected at least three groups in the nonoverlapping centroid areas from each of the clusters (five samples belonged to cluster 1, which was divergent from the other clouds and showed a much more scattered point distribution). Consequently, the physicochemical and microbiological characterizations (cultivable microbiota and 16S rRNA sequencing) for each sample were performed at two different time points corresponding to the winter and summer seasons. Based on this analysis, we selected 23 dairy farms for which specific per-company farming practices and herd characteristics have been reported (see Table S5).

### Winter versus summer milk compositional analysis.

The milk sample subset from the 23 dairy companies was evaluated in terms of physicochemical parameters (Table 1). Notably, the winter samples were significantly enriched (*P* < 0.001) for somatic cells (485.38 ± 299.58 SC/mL), BHBA (0.06 ± 0.02 mmol/liter), and citric acid (0.12% ± 0.01%) compared with the summer samples (114.89 ± 60.90 SC/mL, 0.02 ± 0.01 mmol/liter, and 0.10% ± 0.01%, respectively). Among the macronutrients, seasonality did not affect the protein and casein distributions at the two sampling points. In contrast, lactose was slightly higher in summer (4.77% ± 0.07%) than in winter (4.67% ± 0.10%); fat content had an opposite trend (4.04% ± 0.31% and 3.73% ± 0.53% in winter and summer, respectively). Moreover, winter samples were enriched in the following fatty acids: (i) myristic acids (0.37% ± 0.031% versus 0.34% ± 0.04%); (ii) palmitic acid (1.01% ± 0.08% versus 0.92% ± 0.13%); (iii) short-chain fatty acids (0.53 ± 0.05 versus 0.48 ± 0.08 g/100 g fat); (iv) medium chain fatty acids (1.57 ± 0.14 versus 1.48 ± 0.19 g/100 g fat); (v) monounsaturated fatty acids (1.14 ± 0.1 versus 1.05 ± 0.15 g/100 g); (vi) saturated fatty acids (2.66 ± 0.21 versus 2.45 ± 0.36 g/100 g fat); (vii) unsaturated fatty acids (1.4 ± 0.18 versus 1.09 ± 0.16 g/100 g fat); and (viii) trans fatty acids (0.07 ± 0.02 versus 0.03 ± 0.02 g/100 g fat). Bearing in mind the assessment of dairy technological traits, winter milk samples showed higher values of H index (0.78 ± 0.032 versus 0.68 ± 0.03 μm), firming time (K20 of 21.86 ± 4.199 versus 6.55 ± 0.95 min) and coagulation time (R of 28.9 ± 2.40 versus 22.98 ± 1.93 min), while summer samples performed better in terms of the consistency of coagulum (A30 of 30.38 ± 3.74 versus 21.86 ± 4.19 mm) (Table 1).

**TABLE 1:** Somatic cell composition and content relative to cow raw milks belonging to dairy farms during winter and summer

Property/characteristic	Seasonal value (mean ± SD)		*P* value^*a*^
	Winter	Summer	
Physicochemical and biological markers			
pH (unit)	6.62 ± 0.03	6.69 ± 0.03	<0.001
Somatic cells (SC/mL)	485.38 ± 299.58	114.89 ± 60.90	<0.001
Acetone (mmol/liter)	0.03 ± 0 0.02	0.04 ± 0.03	0.028
BHBA (mmol/liter)	0.06 ± 0.02	0.02 ± 0.01	<0.001
Urea (mg/dL)	24.21 ± 6.01	22.78 ± 5.36	NS
Citric acid (%)	0.12 ± 0.01	0.10 ± 0.01	0.046
Electrical conductivity	862.61 ± 42.07	871 ± 33.03	NS
Crio	−527.33 ± 9.03	−528.64 ± 4.35	NS
Carbohydrates			
Lactose (%)	4.67 ± 0.10	4.77 ± 0.07	<0.001
Proteins			
Protein (%)	3.58 ± 0.20	3.53 ± 0.18	NS
Casein (%)	2.85 ± 0.18	2.78 ± 0.15	NS
Fats			
Fat (%)	4.04 ± 0.31	3.73 ± 0.53	0.024
Myristic acid (%)	0.37 ± 0.03	0.34 ± 0.04	0.017
Oleic acid (%)	1.19 ± 0.11	1.10 ± 0.16	NS
Stearic Acid (%)	0.35 ± 0.04	0.34 ± 0.06	NS
Palmitic acid (%)	1.01 ± 0.08	0.92 ± 0.13	0.004
SCFA (C_4_–C_6_) (g/100 g milk)	0.53 ± 0.05	0.48 ± 0.08	0.012
MCFA (C_8_–C_15_) (g/100 g milk)	1.57 ± 0.14	1.48 ± 0.19	0.041
LCFA (C_16_–C_18_) (g/100 g milk)	1.48 ± 0.16	1.37 ± 0.22	NS
MUFA (C_18:1_) (g/100 g milk)	1.14 ± 0.10	1.05 ± 0.15	0.035
PUFA (g/100 g milk)	0.12 ± 0.02	0.11 ± 0.01	NS
Saturated FA (g/100 g milk)	2.66 ± 0.21	2.45 ± 0.36	0.018
Unsaturated FA (g/100 g milk)	1.4 ± 0.183	1.09 ± 0.16	<0.001
Trans FA (g/100 g milk)	0.07 ± 0.023	0.03 ± 0.02	<0.001
Solids			
Total solids (%)	12.97 ± 0.50	12.68 ± 0.57	NS
Solids nonfat (%)	8.83 ± 0.27	8.95 ± 0.24	0.047
Clotting characteristics			
Consistency of coagulum A30 (mm)	21.86 ± 4.12	30.38 ± 3.74	<0.001
Firming time K20 (min)	7.95 ± 0.97	6.55 ± 0.95	<0.001
Coagulation time R (min)	28.9 ± 2.40	22.98 ± 1.93	<0.001

a BHBA, β-hydroxybutyric acid; SCFA, short-chain fatty acids; MCFA, medium-chain fatty acids; LCFA, long-chain fatty acids; MUFA, monounsaturated fatty acids; PUFA, polyunsaturated fatty acids; FA, fatty acids. NS, not statistically significant (*P *> 0.05).

### Cultivable microbiota from winter and summer milk samples.

The 23 raw milk samples analyzed once in summer and once in winter were inspected for their cultivable microbiota content (Table 2). In detail, the total mesophilic aerobic microorganism cell densities were higher (*P < *0.001) in winter (5.99 ± 1.60 log CFU/g) than in summer (4.55 ± 0.77 log CFU/g). Presumptive mesophilic and thermophilic lactobacilli cell densities in winter were 3.02 ± 0.73 and 2.43 ± 0.54 log CFU/g, respectively. In summer, cell densities for both of these bacterial groups significantly decreased (*P < *0.001) until reaching cell densities of 2.33 ± 0.64 and 1.61 ± 0.33 log CFU/g, respectively. No significant differences were retrieved for culturable presumptive mesophilic and thermophilic cocci, total coliforms, Staphylococcus, Pseudomonas, and yeasts. Additionally, sample cell densities relative to *Enterobacteriaceae* were higher in winter samples (3.15 ± 1.04 log CFU/g) than in those collected during the summer (2.27 ± 0.89 log CFU/g).

**TABLE 2:** Cell densities compared to the principal microbial group evaluated in winter and summer Apulian raw milks

Microbial group	Log CFU/mL		*P* value^*a*^
	Winter	Summer	
Total mesophilic aerobic microorganisms	5.99 ± 1.60	4.55 ± 0.77	<0.001
Mesophilic lactobacilli	3.02 ± 0.73	2.33 ± 0.64	<0.001
Thermophilic lactobacilli	2.43 ± 0.54	1.61 ± 0.33	<0.001
Mesophilic cocci	3.96 ± 0.59	4.17 ± 0.87	NS
Thermophilic cocci	3.74 ± 0.67	3.73 ± 0.73	NS
Total coliforms	2.45 ± 1.01	2.06 ± 0.98	NS
Escherichia coli	0.54 ± 0.87	0.43 ± 0.74	NS
*Enterobacteriaceae*	3.15 ± 1.04	2.27 ± 0.89	0.001
Staphylococcus	1.38 ± 1.13	0.80 ± 1.23	NS
Pseudomonas spp.	2.41 ± 2.10	2.14 ± 1.48	NS
Yeasts	1.25 ± 1.16	1.84 ± 0.99	NS

a NS, not statistically significant (*P *> 0.05).

### Microbiota characterization by 16S rRNA gene high-throughput amplicon sequencing.

To deeply inspect milk microbiota, a targeted high-throughput sequencing analysis based on the V1-V3 hypervariable regions of the 16S rRNA gene was carried out on 23 raw cow milk samples, in both the winter and summer seasons. Demux filtering statistics showed that the number of total input reads ranged from 17,000 to 112,000, whereas the total denoised deblur reads ranged from 7,000 to 46,000 (data not shown). Chimeric, hit-artifact, and missed-reference reads were negligible. Neither Shannon nor Faith’s phylogenetic diversity (PD) indices revealed statistically significant differences in alpha diversity values for winter versus summer milk samples, whereas the beta diversity computed by using the dedicated Emperor plugin within the QIIME2 pipeline allowed us to distinguish them in two different and only partially overlapping clouds (see Fig. S3a in the supplemental material). The relative pairwise permutational multivariate analysis of variance (ANOVA) test indicated that the two groups significantly differed (see Fig. S3b to d).

### Winter core microbiome.

**FIG 1:**
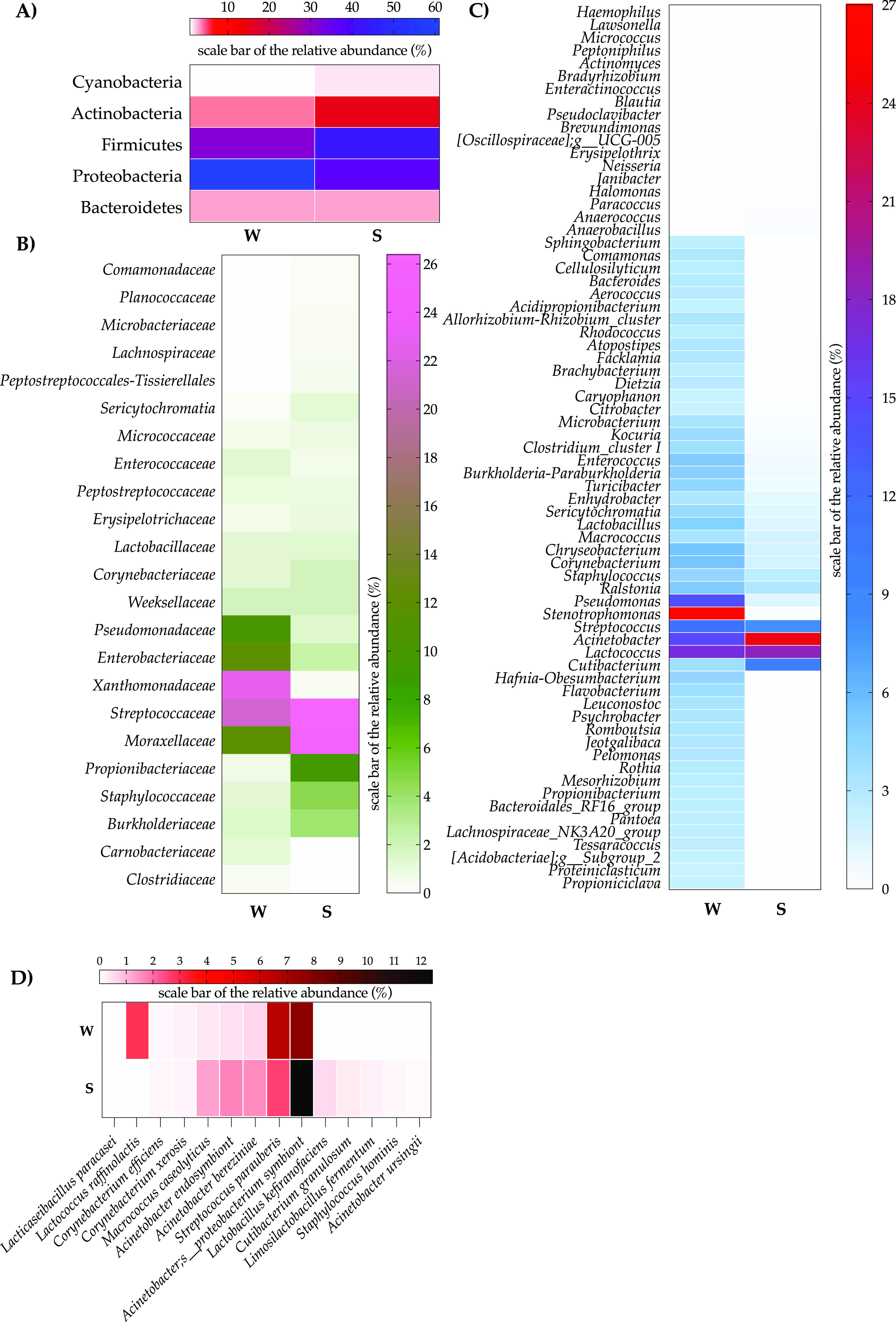
Seasonal core microbiota relative abundance levels (as percentages) at the phylum (A), family (B), genus (C), and species (D) taxonomic levels for raw cow milk samples in winter (W) and summer (S).

A derived microbiota taxa matrix was obtained by maintaining those taxa with a relative abundance of >0.1% and a prevalence of >50%. As a result, the taxonomic investigation highlighted how 5 out of 18 assigned phyla were harbored by the constitutive core microbiota of winter milk samples (Fig. 1A). More specifically, *Proteobacteria* (60.9%) and *Firmicutes* (31.1%) dominated the winter microbiota, whereas *Actinobacteria* (3.8%), *Bacteroidetes* (2.9%), and *Cyanobacteria* (0.9%) accounted for a relative abundance of <10%. According to the fixed criteria, winter samples were populated by 18 families of core microbiota (Fig. 1B). Five bacterial families showed an average relative abundance of >10%: *Xanthomonadaceae* (22.67%), *Streptococcaceae* (21.2%), *Enterobacteriaceae* (11.8%), *Moraxellaceae* (11.9%), and *Pseudomonadaceae* (10.4%). The remaining families, with a relative abundance ranging from 0.1% to 2.02%, were classified as satellites instead (Fig. 1B). At the genus and species levels, the abundance detection threshold that we used was lowered to 0.001%, whereas the prevalence remained the same. Consequently, 47 bacterial genera were included, 19 of which had a prevalence ranging between 80% and 100%. Notably, the genera *Stenotrophomonas* (22.6%), *Lactococcus* (13.3%), Acinetobacter (11.4%), Pseudomonas (10.4%), and Streptococcus (7.9%) had a relative abundance of >5% (Fig. 1C). At the species level, 9 taxa comprised the core microbiome (Fig. 1D), where Acinetobacter proteobacterium symbiont (7.7%), Streptococcus uberis (6.4%), and Lactococcus raffinolactis (2.9%) were the species accounting for the highest relative abundances, while the other 6 identified species did not reach the 1% relative abundance level (Fig. 1D).

### Summer core microbiota as estimated by high-throughput sequencing.

The above-described criteria were used to assess the core microbiota in milk collected during summer at the phylum level. *Firmicutes* (42.9%), *Proteobacteria* (37%), and *Actinobacteria* (15.2%) exhibited the highest abundances, whereas *Bacteroidetes* (2.8%) and *Cyanobacteria* (1.5%) abundances were lower than the threshold of 10% (Fig. 1A). A total of 21 bacterial families were harbored by the core microbiota, with *Streptococcaceae* (26.3%), *Moraxellaceae* (25.8%), *Propionibacteriaceae* (9.8%), *Staphylococcaceae* (4.7%), and *Burkholderiaceae* (4%) having the highest percentage values (Fig. 1B). Forty-six genera comprised the core microbiota in summer samples (Fig. 1C). Twenty-six of the 46 genera showed a prevalence higher than 80%. According to the 5% threshold of relative abundance, Acinetobacter (24.1%), *Lactococcus* (18.3%), *Cutibacterium* (9.0%), and Streptococcus (8.0%) were labeled the major contributing genera. At the species level, the core microbiota included 5 dominant taxa, i.e., Acinetobacter proteobacterium symbiont, which is hierarchically assigned to the Acinetobacter genus (12.4%), Streptococcus parauberis (2.60%), Acinetobacter endosymbiont (1.64%), Acinetobacter bereziniae (1.60%), and Macrococcus caseolyticus (1.27%), whereas the remaining 7 satellite species did not reach the fixed relative abundance threshold of 1% (Fig. 1D).

### Winter versus summer dominant and subdominant microbiomes.

**FIG 2:**
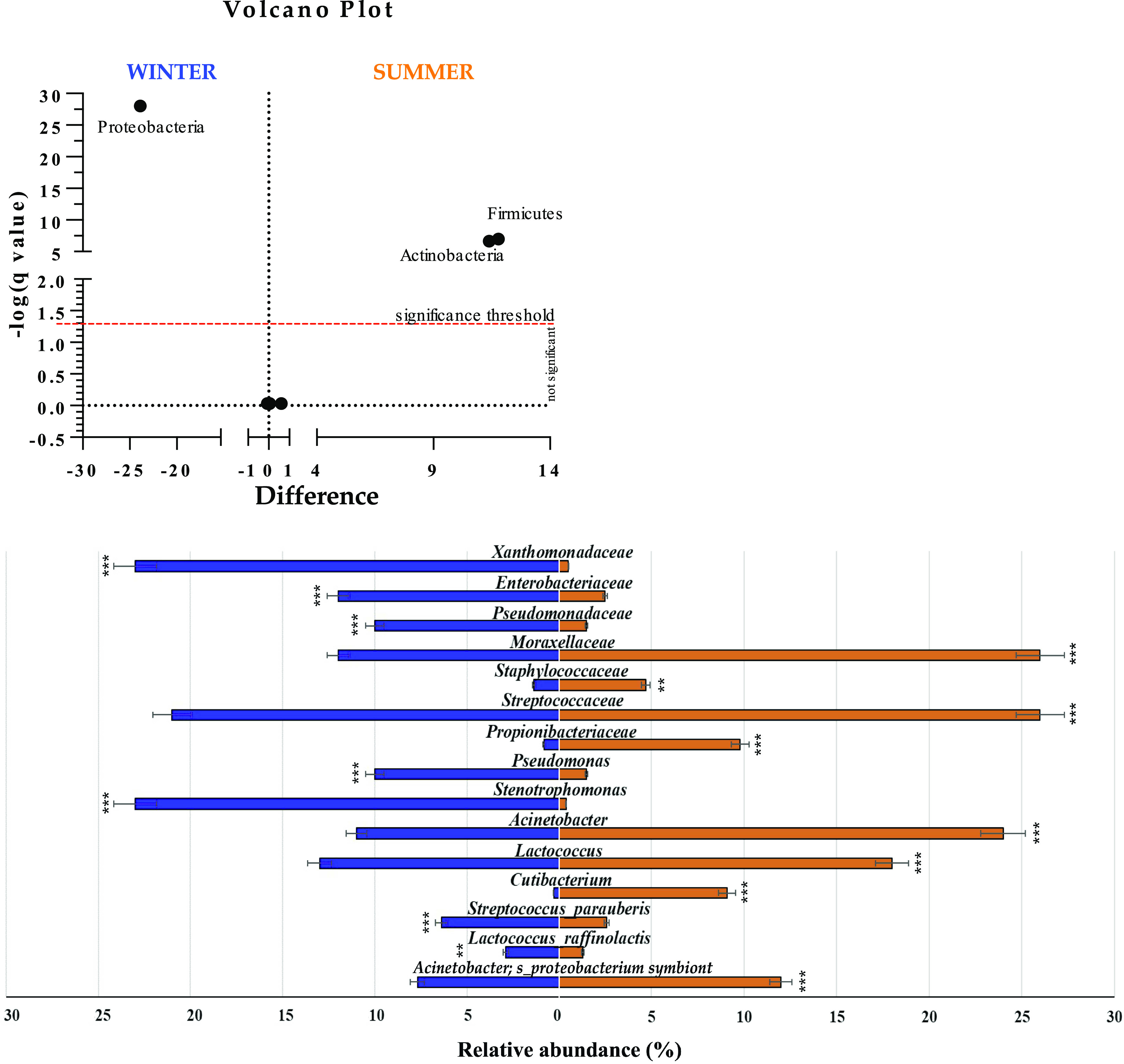
Highest and lower microbiome taxa in raw cow milk samples split by seasons. Microbial differences are reported at the phylum (A) and other taxonomic levels (families, genera, and species) (B). ***, *q* value < 0.001; **, *q* value = 0.007.

Milk microbiota derived from the same Apulian farms can be distinguished based on seasonality. The winter and summer samples differed in several genera, including *Proteobacteria*, *Firmicutes*, and *Actinobacteria* (Fig. 2A). An abundance of *Proteobacteria* characterized the winter samples (*q* value < 0.001). In contrast, *Firmicutes* and *Actinobacteria* were mainly associated with summer (*q* value < 0.001). For deeper profiling, the enrichment of winter samples in *Proteobacteria* was determined by a cumulative contribution of *Xanthomonadaceae* (22.6%), *Enterobacteriaceae* (11.7%), and *Pseudomonadaceae* (10.37%). In fact, each of these bacterial families had higher values (*q* value < 0.001) in winter than in the summer season. The *Proteobacteria* phylum, mainly comprised of *Moraxellaceae*, was instead higher in terms of abundance during the summer season (*q* value < 0.001). The noticed difference in the *Firmicutes* percentage during summer was markedly determined by *Staphylococcaceae* (*q* value = 0.007) and *Streptococcaceae* (*q* value < 0.001). Additionally, *Propionibacteriaceae* increased (*q* value < 0.001) in the summer season, reflecting the same trend as *Actinobacteria*. Among the genera, Pseudomonas and *Stenotrophomonas* had higher values (*q* value < 0.001) in winter-collected milk. In contrast, Acinetobacter (24.13%), *Lactococcus* (18.31%), *Cutibacterium* (9.08%), *Ralstonia* (3.08%), and Staphylococcus (2.8%) significantly increased in summer (*q* value < 0.001). At the species taxonomic level, Streptococcus parauberis and Lactococcus raffinolactis exhibited a statistically significant increase in winter (*q* value < 0.001). The abundance of a species belonging to the genus Acinetobacter (classified as a Acinetobacter proteobacterium symbiont) markedly characterized the summer samples (*q* value < 0.001) (Fig. 2B). Additionally, owing to their absence in winter, the four other bacterial species were higher in summer, specifically, Limosilactobacillus fermentum (*P* = 0.004), Cutibacterium granulosum (*P* = 0.008), Staphylococcus hominis (*P* = 0.015), and Acinetobacter ursingii (*P* = 0.017) (see Fig. S4).

### Correlation between milk physicochemical properties and the microbiome.

**FIG 3:**
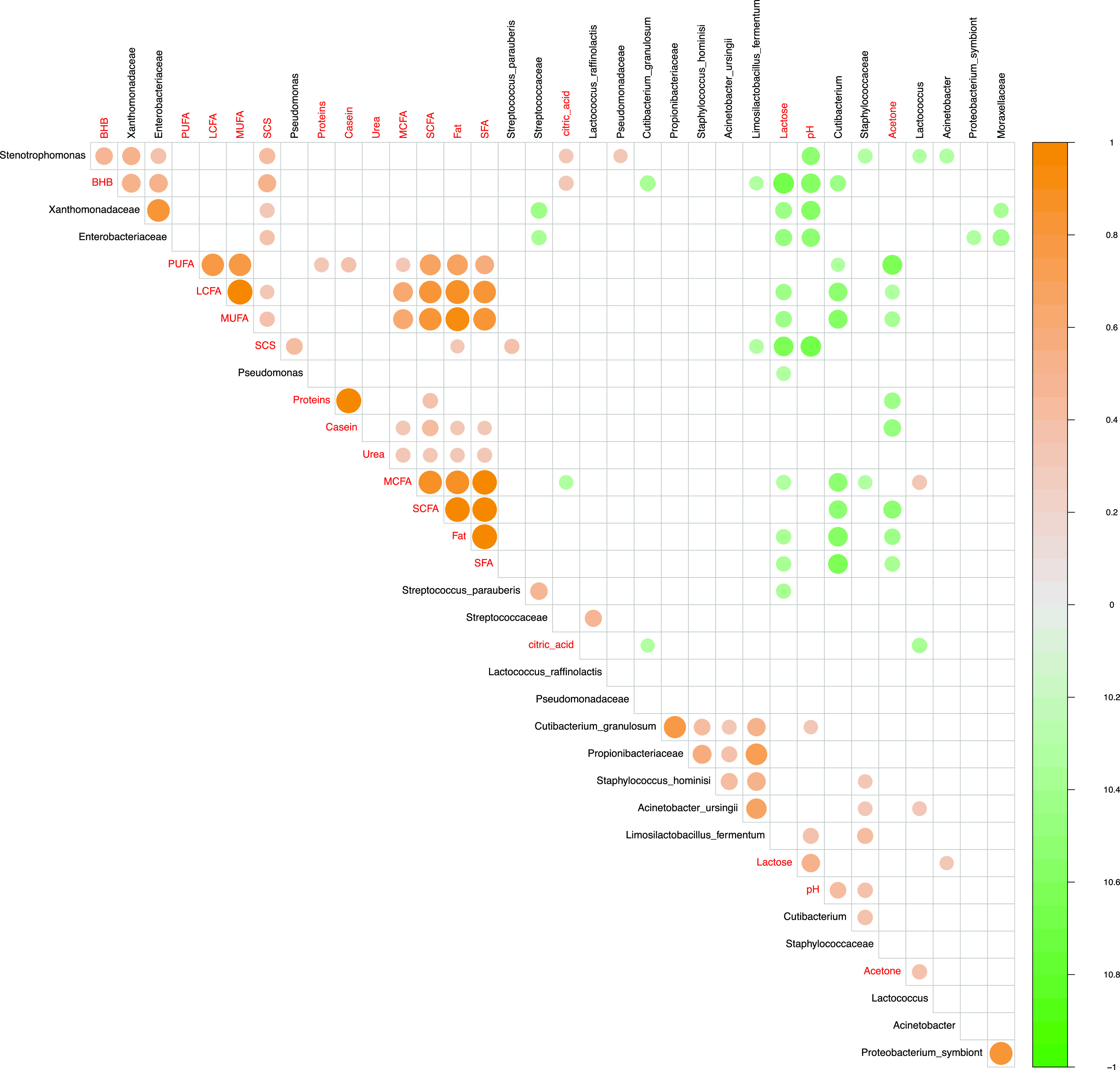
Pearson correlation between milk physicochemical parameters and significantly abundant microbiome taxa (families, genera, and species) between winter and summer raw cow’s milk samples. Large and small circles indicate strong and weak correlations, respectively. Scale bar colors describe the type of correlation: 1 indicates a perfect positive correlation (orange), and −1 indicates a perfect negative correlation (green). BHB, β-hydroxybutyric acid; PUFA, polyunsaturated fatty acid; LCFA, long-chained fatty acid; MUFA, medium unsaturated fatty acid; MCFA, medium-chained fatty acid; SCFA, short-chained fatty acid; SFA, saturated fatty acid; SCS, somatic cells.

Statistically significant differences in the compositional and microbiological profiles depending on seasonality were obtained with a Pearson’s correlation test by comparing winter versus summer samples. Overall, 123 statistically significant correlations were found. Considering those correlations with an absolute *r*^2^ value ranging from 0.3 to 0.5, 43 positive and 32 negative correlations were detected (Fig. 3). A strong correlation was found between *Enterobacteriaceae* and *Xanthomonadaceae* and between these two taxa and the bovine ketosis marker β-hydroxybutyric acid (*r*^2^ > 0.5). Moreover, *Xanthomonadaceae* was positively correlated with the *Stenotrophomonas* genus. Positive correlations were also detected between various taxa, i.e., *Propionibacteriaceae*, Cutibacterium granulosum, Staphylococcus hominis, Acinetobacter ursingii, and Limosilactobacillus fermentum. A strong negative correlation value was found for two comparisons: (i) lactose versus β-hydroxybutyric acid and (ii) pH versus *Stenotrophomonas*, β-hydroxybutyric acid, *Enterobacteriaceae*, *Xanthomonadaceae*, and somatic cells (*r*^2^ < −0.5). Finally, *Cutibacterium* was negatively correlated with many fat constituents, such as long-chain fatty acids (LCFAs), medium unsaturated fatty acids (MUFAs), short-chain fatty acids (SCFAs), fats, and saturated fatty acids (SFAs).

### Differences in metabolic predicted pathways by PICRUSt analysis.

**FIG 4:**
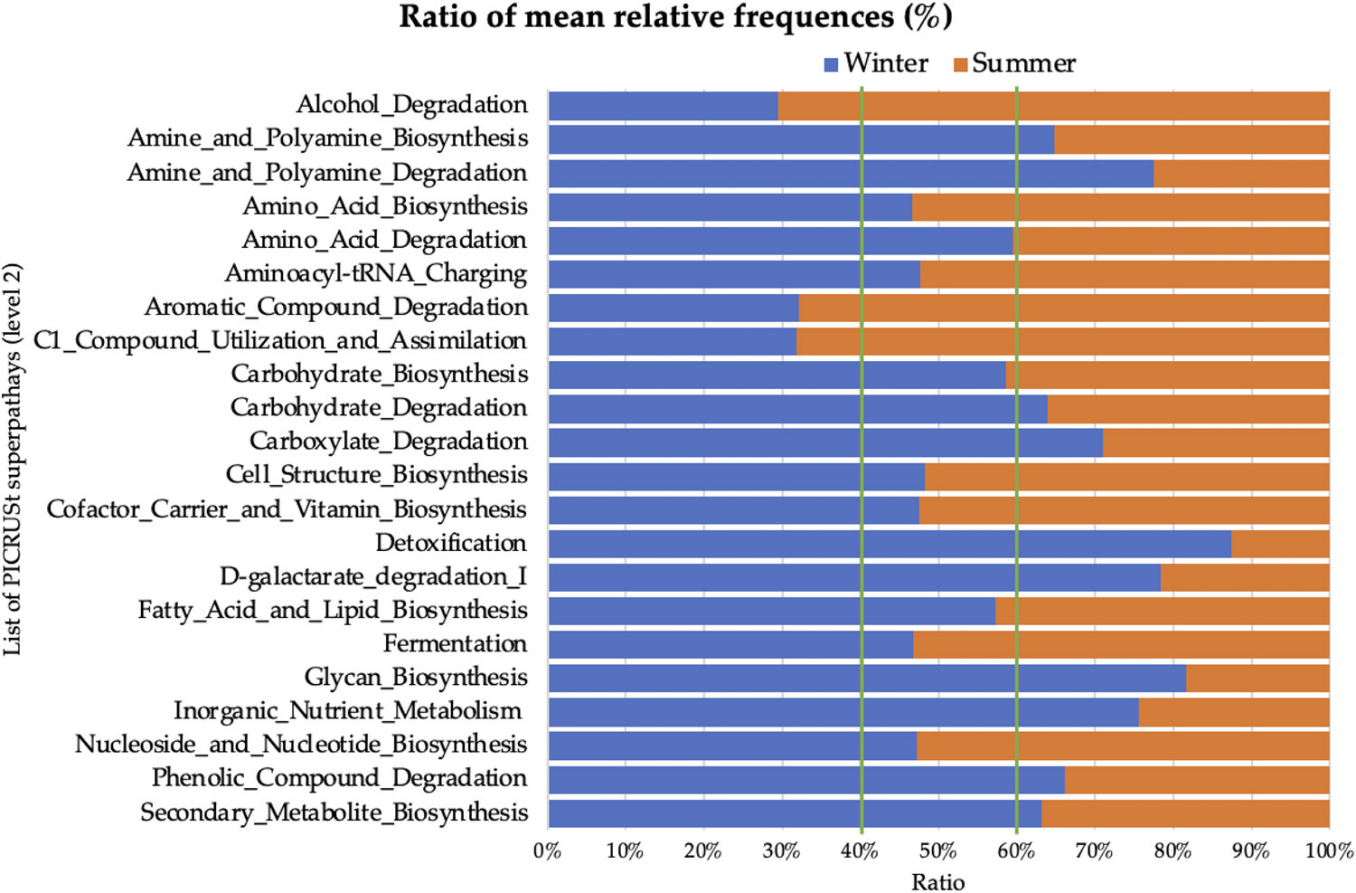
Summer and winter ratios of PICRUSt2-predicted pathways grouped into 22 high-hierarchy levels (second level in the KEGG/Biocyc database). The complete list of single and ungrouped statistically significant metabolic pathways to the lowest level of PICRUSt prediction is reported in Table S6 in the supplemental material.

Starting from 16S rRNA taxa abundances, we predicted the metabolic pathways by using the PICRUSt2 pipeline, and the resulting matrix was inspected to determine statistically significant differences in summer versus winter seasons. Then, to graphically render this high number of predicted pathways, we grouped them considering the second level of a metabolic pathway’s architecture (Fig. 4). Summer samples were enriched in pathways belonging to alcohol degradation, aromatic compound degradation, and C_1_ compound utilization and assimilation. In contrast, the relative frequencies of amine and polyamine biosynthesis and degradation, carbohydrate degradation, carboxylate degradation, detoxification, d-galactarate degradation I, glycan biosynthesis, inorganic nutrient metabolism, phenolic compound degradation, and secondary metabolite biosynthesis were higher in the winter season.

**FIG 5:**
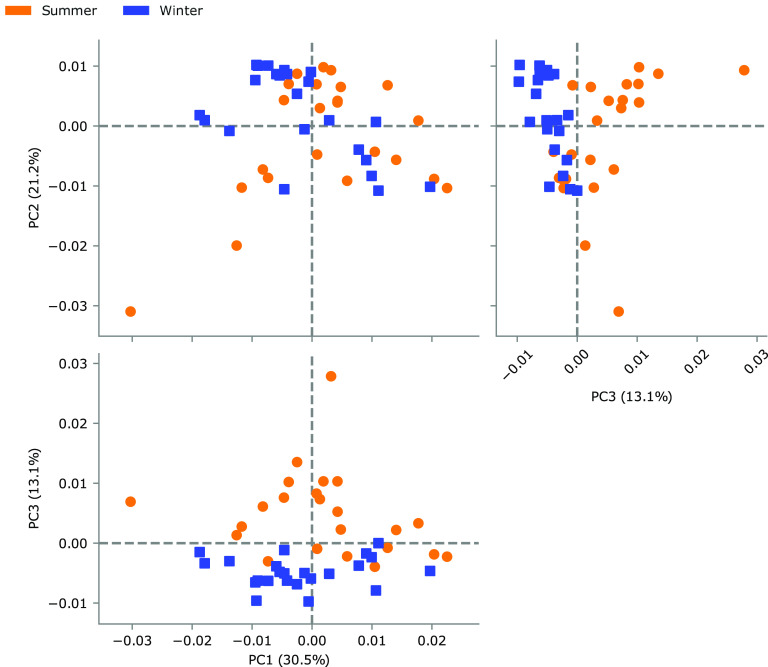
Principal component analysis of winter (blue) versus summer (orange) sample metabolic pathways.

The predicted pathway abundance matrix was used as input for a multivariate statistical analysis (PCA) whose principal components were plotted, allowing us to discriminate samples by seasonality. More specifically, the best cluster separation was obtained by combining the contribution of the third principal component (Fig. 5), which accounted for 13.1% of the total variance.

## DISCUSSION

As the convergent result of a multitude of abiotic and biotic factors having a stochastic, deterministic, and temporary impact on the microbiota (2, 3, 15, 20), milk has been recognized as one of the most complex and unpredictable food matrices.

Here, 638 Apulian dairy farms were profiled to collect information on breeding as well as animal and milk management. These stochastic factors have been recognized as drivers of milk microbiota (2, 3). Milk samples from the same farms were collected and chemically characterized. This allowed us to obtain a wide perspective on the variability characterizing the Apulian cow raw milks. The distribution for fat and protein class content exhibited a high variability in terms of interquartile ranges (21), which may result from a combined contribution of genetic differences and an equal sample number relative to the three analyzed Apulian breeds (i.e., Holstein Friesians, Brown Swiss, Simmenthal). Without superimposing any *a priori* clustering condition, we randomly selected a subset of 23 milk samples from the BIC curve that we considered representative of the total of considered Apulian farm production. This simplified and nonredundant subset allowed us to reduce the differences determined by physicochemical variables, including protein and fat content, as well as changes driven by animal feeding and breeding management. Considering those variables that markedly influenced the environmental and breast microbiota, sampling was performed in the two opposite seasons with the highest differences in terms of humidity (water) and temperature. Pedoclimatic Apulian conditions with hot summers reduce the availability of fresh grass used in dairy herd feeding and inevitably modify grazing habits (22, 23). Therefore, the time spent by cows on pasture was generally higher (26%) during winter than during summer (18%), with an augmented possibility of free grazing. In comparison with extensive farming areas with economically relevant productions, the Apulian region is marked by temperate seasons, typical of the Mediterranean region, fostering the grazing possibility during the cold season. In contrast, the high summer temperatures are contrasted with specific strategies used to reduce the impact of heat stress.

Our data revealed how these habits in turn impact summer and winter milk sample microbiota, which are different in terms of composition and relative abundances. Although *Proteobacteria* and *Firmicutes* dominated the microbiota of milk in both seasons, the former phylum was significantly increased during the winter season. The 16S rRNA gene-based metataxonomic analysis showed that the families *Xanthomonadaceae*, *Enterobacteriaceae*, and *Pseudomonadaceae* constituted the winter milk core microbiota. Genera and species belonging to *Stenotrophomonas* sp., Escherichia coli, Klebsiella pneumoniae, Klebsiella oxytoca, Enterobacter aerogenes, and Pseudomonas aeruginosa are commonly recognized as environmental (e.g., from grazing, bedding, flooring, and stable sources) mastitis pathogens (24–30). Not surprisingly, these three families were positively correlated with somatic cell counts, suggesting potential pathobiont activity. A higher incidence of potential mastitis during winter was associated with climate conditions (31, 32). With this in mind, we can hypothesize that the higher abundance of these pathobionts during winter is linked to the possible onset of mastitis. From this perspective, water and medium-low temperatures that specifically characterize Apulian winter climatic conditions would favor their growth. These findings are in line with previously published studies arguing that mean temperatures ranging from *T*_min_ 5°C to *T*_max_ 11°C (with a minimum and maximum registered peak temperature of 0°C and 20°C, respectively) (33) and constant rainfall (increased humidity) during the cold season favor the growth of pathobionts (34–37). Additionally, the outdoor system (61% of interviewed farms) and pasture time during the cold season may drive the microbial taxa balancing on cow teats, milking parlors, and bedding surfaces (8, 20). Specifically, while grazing, cattle may come into contact with microorganisms that typically belong with soil and plants, i.e., *Xanthomonadaceae*, *Pseudomonadaceae*, and *Enterobacteriaceae* (38–40), which may be later transferred to bedding surfaces. The higher abundances of these taxa were in agreement with the inspected metabolic pathways of fatty acid (FA) and lipid biosynthesis, which were higher during the winter season. The increased expression of unsaturated FAs, such as oleate, dodec-5-enoate, and palmitoleate and its derived *cis*-vaccenate biosynthesis pathway, highlighted the potential role of homeoviscous adaptation of the above-mentioned taxa to colder temperatures (41, 42). It follows that the presence of *Enterobacteriaceae* may counteract the indigenous milk antimicrobial agent lactoferrin, which reduces iron bioavailability by increasing chorismate biosynthesis (43). This molecule acts as a precursor for the biosynthesis of the siderophore enterobactin (44, 45), which was significantly higher, together with chorismate, in our winter milk samples. In a dependent manner, the synergistic *Pseudomonadaceae* proteolytic activity increased when iron was at a growth-limiting concentration (46). Altogether, many superpathways related to carbohydrate, carboxylate, amino acid, amine, and polyamine degradation were also found to be more abundant in winter raw milk. This evidence suggested a greater predisposition of milk to spoilage induced by *Xanthomonadaceae*, *Enterobacteriaceae*, and *Pseudomonadaceae*, whereas their presence negatively affected the derived dairy qualities (38). Due to the discoloration they cause and the proteinase and lipase activities, many species within the *Pseudomonadaceae* family, i.e., Pseudomonas fluorescens, Pseudomonas fragi, Pseudomonas putida, and Pseudomonas ludensis, have been recognized to be the most bitter enemies of milk hygiene and safety and the primary cause of milk alteration (47, 48). Moreover, due to their fermentative activities and the resulting gas and biogenic amine production (13–15, 49), other genera (i.e., Acinetobacter, *Listeria*, *Serratia*, *Clostridium*, and *Chryseobacterium*) are also known for their negative effects on raw milk-derived dairy products (13–15).

In contrast, the shift toward a higher presence of *Firmicutes* and *Actinobacteria* in the summer season is in line with the increased abundances that we found for the dominant core families of *Moraxellaceae*, *Streptococcaceae*, *Propionibacteriaceae*, and *Staphylococcaceae*. Although the higher temperatures during the summer season would favor the growth of *Moraxellaceae* and *Staphylococcaceae* (50, 51), the species belonging to these taxa accounting for Acinetobacter proteobacterium symbiont and Staphylococcus hominis do not constitute cause of concern toward dairy quality because of pasteurization and good hygiene manufacturing practices (2, 52). Instead, the *Propionibacteriaceae* and especially *Streptococcaceae* families include, at lower taxonomic levels, many genera and species that are important to maintain quality. In line with this, our data revealed a significantly higher content of *Lactococcus* during summer. According to our results, Garroni and colleagues hypothesized that LAB may benefit from the higher temperatures in warmer seasons (53). In addition, our data showed a significantly higher abundance of *Limosilactobacillus fermentum* in summer milk. In agreement, other studies have reported an enrichment of LAB genera during the hot season (15, 20, 54).

An increase in pathways related to amino acid biosynthesis and fermentation may be related to a greater presence of LAB that are commonly used to provide the typical cheese flavor (2, 20). Indeed, *Lactococcus* and *L. fermentum* constitute the core microbiota of many natural starters and cheeses (2) and are important producers of many sensorial compounds that ensure cheese qualities. *L. fermentum* typifies the main composition of natural whey cultures used to produce many traditional cheeses in southern Italy, such as Caciocavallo Pugliese (55), Caciocavallo Silano (56), and Caciotta (57). On the other hand, a limited role has been attributed to the presence of this obligately heterofermentative LAB in Grana production (Parmigiano Reggiano and Grana Padano). Its presence could interfere with a grainy texture by forming microholes. Nevertheless, molecular methods have demonstrated the presence of lysed cells of this LAB species in Grana, leading to the release and activation of intracellular enzymes (58). As a consequence, the effect of this process may affect cheese texture and flavor (59). Moreover, the hot season enhances the greater presence of these pro-technological microorganisms linked to milk preservation (9). Indeed, many LABs may induce the production of bacteriocin, organic acids, diacetyl, and hydrogen peroxide, which counteract spoilage through the inhibition of many food-spoiling microorganisms (16–19). Given the importance of milk hygiene and its preservation, an increased presence of LAB is considered of relevant interest for cheesemakers, whereas many autochthonous LAB and non-starter LAB may compensate or complement the use of primary starters, ensuring diversity among cheese varieties and enhancing dairy biodiversity (5, 15, 60).

In this study, we carefully inspected the main biotic and abiotic variables impacting Apulian raw milk. Despite the fragmentation in terms of herd numbers, the Apulian dairy farm management results are homogeneous for many factors that act by reducing the possible cross-contamination within farm indoor activities (e.g., milking). On the other hand, microbiological variability due to seasonal fluctuations was observed as the result of different microbial growth conditions (e.g., humidity, rain activity, and temperature) and finally dependent on some managerial choices, such as grazing, feeding, outdoor versus indoor time, and seasonal pattern birthing. In this sense, the winter raw cow milk harbored many microorganisms, such as *Xanthomonadaceae*, *Enterobacteriaceae*, and *Pseudomonadaceae*, whose presence may have led to a pathobiont microbial niche, worsening the udder and teat tissue conditions and potentially weakening the raw cow milk quality over time for further processing. In contrast, the summer season seemed to provide advantages related to growth of microorganisms of veterinary interest (*Moraxellaceae* and *Staphylococcaceae*). The higher presence of *Streptococcaceae* (i.e., *Lactococcus*) and *Limosilactobacillus fermentum* (core dominant microbiota) in summer milk could be potential positive agents useful in better biopreserving raw milk quality but, on the other hand, constitute a reservoir of pro-technological LAB for further dairy processing.

## MATERIALS AND METHODS

### Questionnaire and dairy farm profiling.

Questionnaire forms were structured with the purpose of collecting the greatest profiling of dairy farms (20); they were delivered to 1,535 dairy farms in the Apulian territory. The questionnaire, with binary answer choices (i.e., 1 for yes and 0 for no), included a total of 16 questions (Q1 to Q16) and collected information about farm management (farm size, number of cattle, hygiene conditions, milking practices, and environment type), breeding type (traditional or organic), animal conditions (breed type, health, animal welfare, housing type), feeding (fodder, pasture, etc.), weaning (e.g., powdered milk), milking, and milk manipulation after collection. By considering only those Apulian farms that fully completed the questionnaire, the total set composed of 1,535 possible farms was restricted to 638 farms.

### Milk sample collection and physicochemical analysis.

During the complete solar year 2019 to 2020 and following the guidelines defined by the Associazione Regionale Allevatori Puglia, we collected raw cow milks from the set of 638 profiled dairy farms. Bulk milk, under constant stirring and maintained at refrigerated conditions (≤4°C), was immediately processed or alternatively stored at −80°C for further analyses. Chemical analyses of milks concerned total proteins, caseins, fats, lactose, urea, acetone, BHBA, solids nonfat, citric acid, saturated FAs (short, medium, and long chain), monounsaturated FA, polyunsaturated FA, and trans FA. Freezing point depression, pH, H index, and electrical conductivity were also measured. Milks were thawed at room temperature (20 to 24°C) and vortexed for 10 s to ensure adequate homogeneity. A volume of 5 mL of sample was warmed at 40°C in a water bath and analyzed by MilkoScan 7 RM (Italian Foss Electric, Padova, Italy) based on Fourier transform infrared spectroscopy technology (2 to 10 μm) (61). To optimize the robustness and accuracy of the analysis, multiple wavelengths selected from the entire mid-infrared spectrum were used for calibration. The MilkoScan 7 RM technique complies with ISO 9622/IDF 141:2013 (62) and Association of Official Analytical Chemists 972.1 official method. The analysis of milk coagulation properties was performed by using the Formagraph instrument (Foss Electric A/S, Hillerød, Denmark) (63). The following three parameters were recorded: curd firmness at 30 min (A30, in millimeters), curd-firming time (K20, in minutes), and rennet coagulation time (R, in minutes). Somatic cell counts (SCC, as SCC per milliliter) were determined with BacSomatic system (Italian Foss Electric, Padova, Italy). To obtain a unique profile for each sample, three biological replicates from each milk were analyzed. The obtained profiles were used to categorize milks. According to the resulting milk profiles, discriminant analysis of principal components (DAPC) was used to evaluate variable contributions to sample clustering. Specifically, the find.clusters function (https://www.rdocumentation.org/packages/adegenet/versions/2.0.1/topics/find.clusters) within the adegenet R package, which offers a robust alternative to Bayesian clustering, was used to subsample the number of screened milks without reducing the variance among samples. In detail, to identify the optimal number of clusters, *k*-means were sequentially run at increasing values of *k*. As a result, different clustering options were evaluated by inspecting the Bayesian information criterion (BIC) curve.

### Cultivable microbiota.

Milk microbiological analyses were performed in agreement with specific standardized methods by an accredited laboratory (Studio Summit S.r.l., Avellino, Italy). The total aerobic counts based on the pour plate technique at 30°C were carried out according to UNI EN ISO 4833-1:2013 (64). The evaluation of total coliforms and β-glucuronidase^+^ E. coli counts was carried out according to AFNOR BIO 12/20-12/06 (65). Staphylococcus coagulase-positive strains and *Enterobacteriaceae* were directly quantified after enrichment by following the respective official methods, ISO 6888-1:2021 (66) and ISO 21528-2:2017 (67). Presumptive Pseudomonas counts were determined by applying ISO/TS 11059:2009 (68). Presumptive lactic acid bacteria were prepared by serial dilutions in Ringer solution and plated on different culture media purchased from Oxoid (Basingstoke, United Kingdom). In detail, presumptive mesophilic lactobacilli and cocci were enumerated using de Man, Rogosa, and Sharpe (MRS) medium and lactose M17 agar plates, respectively. Both media were supplemented with cycloheximide (0.1% [wt/vol]) and incubated at 30°C. Presumptive thermophilic lactobacilli and streptococci were enumerated on MRS and lactose M17 agar media, respectively. Both media were supplemented with cycloheximide (0.1% [wt/vol]) after incubation at 45°C (69). Enumeration of yeasts and molds was assessed by means of the selective medium Dichloran Rose-Bengal chloramphenicol agar (Biolife) in accordance with ISO 21527-1:2008 (70).

### Extraction and sequencing of total bacterial DNA.

Milk DNA was extracted using the FastDNA spin kit (MP Biomedicals, Solon, OH, USA) following the manufacturer’s instructions. A negative control for DNA extraction was also included to guarantee the identification of potential kit contamination (71). The quality and concentration of total extracted DNA were evaluated spectrophotometrically (NanoDrop ND-1000; Thermo Fisher Scientific, Inc.). 16S rRNA metagenomic sequencing was carried out by Genomix4life (a spinoff of the University of Salerno, Fisciano, Italy) on an Illumina MiSeq platform. In detail, the primers used for the target sequence were 28F (forward, 5′-GAGTTTGATCNTGGCTCAG-3′) and 519R (reverse, 5′-TGCTGCCTCCCGTAGGAGT-3′), allowing the amplification of the V1-V3 hypervariable region of the 16S rRNA marker gene, according to the Genomix4life internal protocols (https://www.genomix4life.com/en/sequencing.html). A negative control for sequencing was also included in the workflow of 16S amplification and library preparation, consisting of all the reagents included in the sample processing and without the sample, to ensure that no contamination took place. Libraries were quantified using a Qubit fluorometer (Invitrogen Co., Carlsbad, CA, USA) and pooled, including the Phix control library, to an equimolar amount (4 nM final concentration). FastQ file quality was assessed by using FastQC software (72) and analyzed by using the QIIME2 dedicated pipeline (https://qiime2.org) microbiome platform (version 2020.8). Denoising was computed with the q2-deblur QIIME plugin (https://github.com/qiime2/q2-deblur). Taxonomy was inferred with the QIIME-compatible database Silva v.138 SSU, using an amplicon sequence variant (ASV) table based on error-corrected reads. Alpha diversity metrics, including Shannon entropy and Faith’s PD, were also computed by using QIIME2 nested plugins (73).

### PICRUSt.

Functional metabolic profile prediction was carried out by using the Phylogenetic Investigation of Communities by Reconstruction of Unobserved States (PICRUSt2) pipeline, version 2.0 (74). Pathway prediction was based on QIIME2 16S rRNA gene meta-barcoding-derived taxonomy in the form of a raw matrix (.biom file). Further clustering of the metabolic pathways was carried out by using Biocyc database pathway assignation.

### Statistical analyses.

The obtained data are expressed as means ± SD, medians with interquartile ranges (IQR; i.e., 25th to 75th percentiles), or percentages, as appropriate. Continuous variables were subjected to one-way ANOVA. In contrast, nonparametric data were analyzed by the rank test using the Wilcoxon Mann-Whitney test corrected for multiple comparisons by the Sidak-Bonferroni method using GraphPad Prism (v. 8.4.0). Metabolic predicted pathways were compared by using a two-sided Welch test corrected for multiple comparisons with the Benjamini-Hochberg correction. Only statistically significant values corrected by multiple tests were considered and discussed.

### Data availability.

All relevant data are included in the main body or the supplemental material associated with the article. 16S rRNA raw data from the study were deposited in the NCBI Sequence Read Archive (SRA; https://www.ncbi.nlm.nih.gov/sra) under accession code PRJNA807332.
