# Supplementary material for: Effect of Seasonality on Microbiological Variability of Raw Cow Milk from Apulian Dairy Farms in Italy
Source: Microbiol Spectr. 2022 Aug 16;10(5):e00514-22. doi: 10.1128/spectrum.00514-22 (PMC9602280; doi:10.1128/spectrum.00514-22)
Supplement: Supplemental file 1 — Supplemental material. Download spectrum.00514-22-s0001.pdf, PDF file, 7.8 MB [file spectrum.00514-22-s0001.pdf]

Table S1. Questionnaire administered to 638 dairy farms, and related percentage values and numerical intervals.

**Farming conditions**		**Percentage**
**1. Type of farming management**	Conventional farming	95
	Organic farming	5
**2. Number of dairy cows**	Less than 50	24
	Beetween 50 and 100	25
	More than 100	51
**3. Cow breed**	Holstein Friesians	45
	Brown Swiss	32
	Simmenthal	10
	Breed Crosses	13
**4. Dairy Cows Housing**	Tie stalls	6
	Mixed	33
	Freestalls	61
**5. Ventilation system inside the farm**	Yes	100
	No	-
**6. Free-range cattle-breeding**	Yes	94
	No	6
**7. Feeding with colostrum after birth**	Yes	22
	No	78
**8. Use of milk substitutes for weaning calves**	Milk	78
	Milk substitutes	22
**9. Winter basic fodder**	Pasture	26
	Grain	35
	Hay	39
**10. Summer basic fodder**	Pasture	18
	Grain	41
	Hay	41
**11. Percentage of total annual fodder (without concentrate) produced in the farm**	Less than 50%	13
	Between 50 and 70%	-
	More than 70%	87
**12. Organic culture certificate**	Yes	82
	No	18
**13. Udder cleaning and pre-milking done regularly**	Yes	92
	No	8

**14. Milking system**	Parlor	95
	Automatic milking system	5
	Manual	-
**15. House care system during lactation**	Tie stall	39
	Freestalls	61
**16. Average milk production per day**	Less than 50 (quintals)	39
	Between 50 and 100 (quintals)	5
	More than 100 (quintals)	56

Table S2. Questionnaire administered to 638 dairy farms, aiming to highlight management, and feeding strategies modified by farmers during the summer.

**Heat stress coping strategies**		**Percentage**
**1. Activation of fun systems in resting and/or feeding area in lactating cow barn (n=638)**	Yes	95
	No	5
**2. Activation of cooling systems in feeding area in lactating cow barn (n=638)**	Yes	58
	No	42
**3. Kind of use of fun and cooling systems in lactating cow barn (n= 606)**	Automatic activation according to environmental THI	86
	Application of cooling protocols	14
**4. Fun/Cooling systems in pre-milking area (n=638)**	Yes	8
	No	92
**5. Fun/Cooling systems in post-milking area (n=638)**	Yes	6
	No	94
**6. Fun/Cooling systems in dry cow barns/close up cows (n=638)**	Yes	4
	No	96
**7. Increase of frequency of daily TMR administration (n=638)**	Yes	68
	No	32
**8. Reduction of TMR humidity (n=638)**	Yes	22
	No	78
**9. Introduction of functional feed additives (yeast products, vitamins, natural immune enhancers, minerals, etc…) (n=638)**	Yes	78
	No	22
**10. Changes of the daily ration (n=638)**	No	55
	Less than 10% of the total ration	36
	From 11% to 50% of the total ration	8
	More than 51% of the total ration	1
**11. Modifications of daily pasture program (n=575)**	No	13
	Increasing grazing time	15
	Decreasing grazing time	72

* TMR: Total mixed ration; THI: temperature-humidity indexes.

Table S3. Physico-chemical composition, technological parameters and somatic cells analysis evaluated on 638 raw cow's milk of Apulian territory.

	median	min	max
**Physico-chemical and biological markers**			
pH (unit)	6.55 ± 0.03	6.32	6.72
Somatic cells SC/ml	261.76 ± 45.30	160.33	309.66
Acetone (mmol/l)	0.04 ± 0.021	0	0.16
β-hydroxybutyric acid (BHBA, mmol/l)	0.07 ± 0.03	0	0.26
Urea (mg/dl)	2.47 ± 4.73	6.77	52.12
Citric Acid (%)	0.11 ± 0.01	0.06	0.2
**Carbohydrates**			
Lactose %	4.73 ± 0.10	4.01	4.99
**Protein components**			
Proteins %	3.5 ± 0.24	2.81	5.03
Casein %	2.73 ± 0.22	2.11	4.45
**Fat components**			
Fat %	3.93 ± 0.67	2.85	9.02
Myristic acid C140 %	0.38 ± 0.06	0.26	0.92
Oleic acid C181 %	1.15 ± 0.18	0.72	2.76
Stearic acid C180 %	0.35 ± 0.06	0.18	0.87
Palmitic acid C160 %	1.03 ± 0.20	0.64	2.68
Short-chain fatty acids (C4-C6) (Scfa, g/100 g milk)	0.51 ± 0.10	0.31	1.25
Medium-chain fatty acids (C8-C15) (Mcfa, g/100 g milk)	1.52 ± 0.28	0.94	3.62
Long-chain fatty acids (C16-C18) (Lcfa, g/100 g milk)	1.45 ± 0.26	0.98	3.66
Mono-unsaturated fatty acids (Mufa, g/100 g milk)	1.11 ± 0.18	0.71	2.65
Poly-unsaturated fatty acids (Pufa, g/100 g milk)	0.12 ± 0.01	0.08	0.23
Saturated fatty acid (Sfa, g/100 g milk)	2.67 ± 0.46	1.63	6.11
Unsaturated fatty acids (Ufa, g/100 g milk)	1.42 ± 0.31	0.67	3.44
Trans fatty acids (Tfa, g/100 g milk)	0.09 ± 0.02	0.03	0.25
**Solid components**			
Solids non-fat (%)	9.07 ± 0.35	8.07	11.38
**Clotting characteristics**			
Consistency of coagulum A30 (mm)	23.55 ± 6.63	7.63	77.16

K20 firming time (min)	8.55 ± 1.26	1	12.33
Coagulation time R (min)	30.22 ± 3.36	13.71	44.1

Table S4. Physico-chemical composition, technological parameters and somatic cells analysis evaluated on 638 raw cow's milk of Apulian territory according to the winter and summer seasons.

	Winter			Summer		
	Median (st.dev)	min	max	Median (st.dev)	min	max
**Physico-chemical and biological markers**						
pH (unit)	6.57 ± 0.04	6.24	6.68	6.53 ± 0.04	6.32	6.71
Somatic cells SC/ml	393.94 ± 370.21	237.61	309.66	245.68 ± 292.97	160.33	225.34
Acetone (mmol/l)	0.05 ± 0.02	0.00	0.17	0.04 ± 0.03	0.00	0.26
β-**hydroxybutyric acid (BHBA, mmol/l)**	0.08 ± 0.03	0.00	0.35	0.07 ± 0.05	0.00	0.31
Urea (mg/dl)	23.92 ± 5.14	4.79	56.75	23.11 ± 5.44	3.27	53.56
Citric Acid (%)	0.11 ± 0.01	0.08	0.20	0.11 ± 0.01	0.06	0.15
**Carbohydrates**						
Lactose %	4.77 ± 0.12	4.12	4.99	4.71 ± 0.13	4.01	4.93
**Protein components**						
Proteins %	3.55 ± 0.23	2.96	5.03	3.39 ± 0.23	2.81	4.73
Casein %	2.78 ± 0.22	2.26	4.45	2.65 ± 0.21	2.11	3.86
**Fat components**						
Fat %	4.06 ± 0.59	3.05	9.02	3.74 ± 0.59	2.85	8.55
Myristic acid C140 %	0.4 ± 0.06	0.29	0.92	0.35 ± 0.06	0.26	0.80
Oleic acid C181 %	1.19 ± 0.19	0.82	2.76	1.09 ± 0.18	0.72	2.63

Stearic acid C180 %	0.36 ± 0.06	0.24	0.87	0.34 ± 0.07	0.18	0.86
Palmitic acid C160 %	1.09 ± 0.2	0.67	2.68	0.97 ± 0.19	0.64	2.36
Short-chain fatty acids (C4-C6) (SCFA, g/100 g milk)	0.55 ± 0.09	0.35	1.25	0.47 ± 0.1	0.31	1.08
Medium-chain fatty acids (C8-C15) (MCFA, g/100 g milk)	1.58 ± 0.26	0.98	3.62	1.47 ± 0.27	0.94	3.25
Long-chain fatty acids (C16-C18) (LCFA, g/100 g milk)	1.49 ± 0.26	1.00	3.66	1.37 ± 0.26	0.98	3.45
Mono-unsaturated fatty acids (MUFA, g/100 g milk)	1.14 ± 0.19	0.78	2.65	1.06 ± 0.18	0.71	2.63
Poly-unsaturated fatty acids (PUFA, g/100 g milk)	0.13 ± 0.02	0.08	0.26	0.11 ± 0.02	0.09	0.23
Saturated fatty acid (SFA, g/100 g milk)	2.82 ± 0.45	1.83	6.11	2.45 ± 0.45	1.63	5.58
Unsaturated fatty acids (UFA, g/100 g milk)	1.55 ± 0.3	0.76	3.44	1.28 ± 0.31	0.67	3.27
Trans fatty acids (TFA, g/100 g milk)	0.08 ± 0.03	0.04	0.25	0.07 ± 0.02	0.03	0.24
**Solid components**						
Solid non-fats (%)	9.28 ± 0.33	8.09	11.38	8.77 ± 0.35	8.07	10.72
**Clotting characteristics**						
Consistency of coagulum A30 (mm)	25.14 ± 6.43	8.13	76.16	23.03 ± 5.97	7.63	62.99
K20 firming time (mins)	8.26 ± 1.19	1	11.98	8.63 ± 2.2	1.00	12.33
Coagulation time R (mins)	29.73 ± 3.14	13.71	43.87	30.82 ± 7.84	17.30	44.11

Table S5. Farming practices and herd characteristics of the 23 selected dairy cow companies in Apulian region.

	Internal Code	7105508	7105940	7106135	7110629	7111647	7120634	7121151	7121313	7121330	7122107	7122154	7122289	7131423	7131429	7210201	7423805	7510401	7530375	7530401	7530471	7530509	7530573	7530612
1. Type of farming management	Conventional farming	✓	✓	✓	✓	✓	✓	✓	✓	✓		✓	✓	✓	✓	✓	✓	✓	✓	✓	✓	✓	✓	✓
	Organic farming										✓													
2. Number of dairy cows	Less than 50							✓								✓	✓					✓	✓	
	Beetween 50 and 100			✓						✓	✓		✓		✓			✓	✓					
	More than 100	✓	✓		✓	✓	✓		✓			✓		✓						✓	✓			✓
3. Cow breed	Holstein Friesians	✓			✓		✓	✓	✓	✓		✓		✓	✓	✓	✓	✓	✓	✓	✓	✓	✓	✓
	Brown Swiss	✓	✓			✓		✓		✓	✓	✓	✓	✓		✓				✓	✓	✓		
	Simmenthal	✓		✓						✓		✓			✓	✓					✓		✓	
	Breed Crosses															✓	✓				✓		✓	
4. Dairy Cows Housing	Tie stalls											✓						✓						
	Mixed	✓								✓	✓				✓	✓						✓		✓
	Freestalls		✓	✓	✓	✓	✓	✓	✓				✓	✓			✓		✓	✓	✓		✓	
5. Ventilation system inside the farm	Yes	✓	✓	✓	✓	✓	✓	✓	✓	✓	✓	✓	✓	✓	✓	✓	✓	✓	✓	✓	✓	✓	✓	✓
	No																							
6. Free-range cattle-breeding	Yes	✓	✓	✓	✓	✓	✓	✓	✓	✓	✓		✓	✓	✓	✓	✓		✓	✓	✓	✓	✓	✓
	No											✓						✓						
7. Feeding with colostrum after birth	Yes	✓						✓		✓	✓									✓				
	No		✓	✓	✓	✓	✓		✓			✓	✓	✓	✓	✓	✓	✓	✓		✓	✓	✓	✓
8. Use of milk substitutes for weaning calves	Milk		✓	✓	✓	✓	✓	✓					✓	✓	✓	✓	✓	✓			✓	✓	✓	✓
	Milk substitutes	✓							✓	✓	✓	✓							✓	✓				
9. Winter basic fodder	Pasture		✓	✓	✓			✓		✓	✓		✓		✓		✓				✓		✓	
	Grain	✓	✓	✓	✓	✓	✓	✓	✓	✓		✓	✓	✓	✓	✓		✓	✓	✓	✓	✓	✓	✓
	Hay	✓	✓	✓	✓	✓	✓	✓	✓	✓	✓	✓	✓	✓	✓	✓		✓	✓	✓	✓	✓	✓	✓
	Pasture							✓		✓	✓				✓		✓							

10. Summer basic fodder	Grain	✓	✓	✓	✓	✓	✓	✓	✓	✓	✓	✓	✓	✓	✓	✓	✓	✓	✓	✓	✓	✓	✓	✓
	Hay	✓	✓	✓	✓	✓	✓	✓	✓	✓	✓	✓	✓	✓	✓	✓	✓	✓	✓	✓	✓	✓	✓	✓
11. Percentage of total annual fodder (without concentrate) produced in the farm	Less than 50%												✓			✓						✓	✓	
	More than 70%	✓	✓	✓	✓	✓	✓	✓	✓	✓	✓	✓		✓	✓		✓	✓	✓	✓	✓			✓
12. Organic culture certificate	Yes	✓	✓	✓	✓			✓	✓	✓			✓	✓	✓	✓	✓	✓	✓	✓	✓	✓	✓	✓
	No					✓	✓				✓	✓												
13. Udder cleaning and pre-milking done regularly	Yes	✓	✓	✓	✓	✓	✓	✓	✓	✓	✓	✓	✓	✓	✓		✓	✓	✓	✓	✓	✓	✓	✓
	No															✓								
14. Milking system	Parlor	✓	✓	✓	✓	✓	✓	✓	✓	✓	✓	✓	✓	✓	✓	✓	✓	✓	✓	✓	✓	✓	✓	✓
	Automatic milking system																							
15. House care system during lactation	Tie stall							✓		✓	✓				✓		✓	✓			✓			
	Free stall	✓	✓	✓	✓	✓	✓		✓			✓	✓	✓		✓			✓	✓		✓	✓	✓
16. Average milk production per day	Less than 50 (quintals)							✓		✓	✓					✓	✓					✓	✓	
	Between 50 and 100 (q)												✓					✓						
	More than 100 (q)	✓	✓	✓	✓	✓	✓		✓			✓		✓					✓	✓	✓	✓		✓
DAPC cluster*		4	1	6	7	3	4	5	2	7	3	5	1	4	1	6	2	5	1	6	2	7	1	3

* The number (1-7) reported in the column is referred to the belonging cluster of the DAPC analysis computed on physico-chemical parameters of milk

Table S6. Welch test comparison of winter and summer mean relative frequencies (%) of predicted pathways (PICRUSt2) clustered at the second level of Biocyc database. Only corrected p-values (q-values) have been reported.

	Winter	Summer	q-values
**Alcohol degradation**			
Glycerol degradation to 1,3-propanediol	0.004	0.010	0.016
**Amine/Polyamine biosynthesis**			
Polyamine biosynthesis I	0.159	0.076	0.007
Arginine and polyamine biosynthesis	0.214	0.127	0.016
Ornithine degradation	0.176	0.045	<0.001
Phenylethylamine degradation	0.122	0.042	<0.001
**Amino acid biosynthesis**			
Chorismate metabolism	0.242	0.146	0.007
L-glutamate and L-glutamine biosynthesis	0.118	0.205	0.026
L-valine biosynthesis	0.593	0.692	0.004
L-isoleucine biosynthesis I (from threonine)	0.593	0.692	0.004
L-lysine biosynthesis III	0.583	0.650	0.011
L-arginine biosynthesis II (acetyl cycle)	0.481	0.586	0.013
L-isoleucine biosynthesis II	0.636	0.731	0.021
L-isoleucine biosynthesis I	0.551	0.616	0.026
L-histidine biosynthesis	0.431	0.508	0.026
L-isoleucine biosynthesis III	0.531	0.613	0.029
Branched amino acid biosynthesis	0.559	0.643	0.013
L-methionine salvage cycle III	0.001	0.011	0.032
**Amino acid degradation**			
L-arginine, putrescine, and 4-aminobutanoate degradation	0.151	0.037	<0.001
L-arginine and L-ornithine degradation	0.151	0.037	<0.001
L-tryptophan degradation to 2-amino-3-carboxymuconate semialdehyde	0.073	0.010	0.001
L-arginine degradation II (AST pathway)	0.141	0.060	0.012
4-hydroxyphenylacetate degradation	0.131	0.056	0.014
Mandelate degradation to acetyl-CoA	0.001	0.005	0.027
Toluene degradation I (aerobic) (via o-cresol)	0.040	0.084	0.027
Mandelate degradation I	0.000	0.001	0.027
Formaldehyde oxidation I	0.049	0.107	0.028
Toluene degradation II (aerobic) (via 4-methylcatechol)	0.040	0.084	0.028

L-valine degradation I	0.000	0.002	0.033
Catechol degradation I (meta-cleavage pathway)	0.033	0.071	0.033
**Aminoacyl-tRNA charging**			
tRNA charging	0.502	0.551	0.017
**Aromatic compound degradation**			
3-phenylpropanoate degradation	0.008	0.018	0.041
**C1 compound utilization/assimilation**			
Formaldehyde assimilation II (RuMP Cycle)	0.067	0.145	0.039
**Carbohydrate_Biosynthesis**			
UDP-glucose-derived O-antigen building blocks biosynthesis	0.305	0.168	<0.001
Colanic acid building blocks biosynthesis	0.261	0.145	0.007
ADP-L-glycero-β-D-manno-heptose biosynthesis	0.135	0.039	<0.001
GDP-mannose-derived O-antigen building blocks biosynthesis	0.220	0.122	0.012
dTDP-N-acetylthomosamine biosynthesis	0.152	0.074	0.016
UDP-N-acetyl-D-glucosamine biosynthesis I	0.541	0.591	0.047
**Carbohydrate degradation**			
Glucose and glucose-1-phosphate degradation	0.332	0.153	0.002
Sucrose degradation IV (sucrose phosphorylase)	0.366	0.206	0.002
L-rhamnose degradation I	0.099	0.035	0.004
Glucose and xylose degradation	0.405	0.287	0.014
**Carboxylate degradation**			
D-galactarate degradation I	0.097	0.027	0.002
D-glucarate degradation I	0.099	0.028	0.002
2-methylcitrate cycle I	0.165	0.092	0.007
**Cell structure biosynthesis**			
Peptidoglycan biosynthesis I (meso-diaminopimelate containing)	0.547	0.618	0.027
UDP-N-acetylmuramoyl-pentapeptide biosynthesis I (meso-diaminopimelate containing)	0.556	0.626	0.033
(Kdo)2-lipid A biosynthesis	0.196	0.055	<0.001
UDP-N-acetylmuramoyl-pentapeptide biosynthesis II (lysine-containing)	0.528	0.620	0.013
Peptidoglycan biosynthesis III (mycobacteria)	0.534	0.615	0.018
**Cofactor/carrier/vitamin biosynthesis**			
Demethylmenaquinol-6 biosynthesis II	0.000	0.001	0.031
Pyridoxal 5'-phosphate biosynthesis and salvage	0.349	0.232	<0.001
Flavin biosynthesis I (bacteria and plants)	0.423	0.494	0.047
NAD biosynthesis II (from tryptophan)	0.095	0.015	0.001
Coenzyme A biosynthesis I	0.510	0.583	0.015
Mono-trans, poly-cis decaprenyl phosphate biosynthesis	0.039	0.134	0.017

Thiazole biosynthesis II (Bacillus)	0.144	0.079	0.023
S-adenosyl-L-methionine cycle I	0.435	0.531	0.026
Biotin biosynthesis II	0.017	0.105	0.002
Thiamin salvage II	0.350	0.441	0.046
**Detoxification**			
Polymyxin resistance	0.117	0.017	0.001
**D-galactarate degradation I**			
D-glucarate and D-galactarate degradation	0.097	0.027	0.002
**Fatty acid /lipid biosynthesis**			
Fatty acid biosynthesis initiation (E. coli)	0.604	0.392	<0.001
Mycolate biosynthesis	0.704	0.508	0.001
Stearate biosynthesis II (bacteria and plants)	0.617	0.466	0.005
Palmitate biosynthesis II (bacteria and plants)	0.646	0.533	0.048
Oleate biosynthesis IV (anaerobic)	0.679	0.500	0.001
Palmitoleate biosynthesis I (from (5Z)-dodec-5-enoate)	0.662	0.483	0.002
Cis-vaccenate biosynthesis	0.765	0.641	0.028
(5Z)-dodec-5-enoate biosynthesis	0.649	0.465	0.001
**Fermentation**			
Mixed acid fermentation	0.491	0.393	0.001
Pyruvate fermentation to propanoate I	0.108	0.282	0.015
Pyruvate fermentation to isobutanol (engineered)	0.726	0.836	0.044
**Glycan_Biosynthesis**			
Enterobacterial common antigen biosynthesis	0.109	0.025	0.001
**Inorganic nutrient metabolism**			
Methylphosphonate degradation I	0.133	0.043	0.002
**Nucleoside/nucleotide biosynthesis**			
5-aminoimidazole ribonucleotide biosynthesis II	0.547	0.646	0.002
5-aminoimidazole ribonucleotide biosynthesis	0.547	0.646	0.002
5-aminoimidazole ribonucleotide biosynthesis I	0.576	0.659	0.006
Pyrimidine nucleobases salvage	0.650	0.749	0.015
**Adenosine nucleotides de novo biosynthesis II**	0.597	0.676	0.023
8-amino-7-oxononanoate biosynthesis I	0.468	0.348	0.032
Adenosine nucleotides de novo biosynthesis I	0.630	0.706	0.028
Inosine-5'-phosphate biosynthesis I	0.509	0.583	0.015
**Adenosine ribonucleotides de novo biosynthesis**	0.606	0.683	0.021
Guanosine ribonucleotides de novo biosynthesis	0.579	0.671	0.001
Inosine-5'-phosphate biosynthesis III	0.504	0.574	0.027

**Phenolic compound degradation**			
Phenylacetate degradation I (aerobic)	0.142	0.073	0.014
**Secondary_Metabolite_Biosynthesis**			
Enterobactin biosynthesis	0.209	0.086	0.002
Aerobactin biosynthesis	0.076	0.007	0.001
**Secondary_Metabolite_Degradation**			
4-deoxy-L-threo-hex-4-enopyranuronate degradation	0.176	0.088	0.002
anhydromuropeptides recycling	0.587	0.432	0.001

Fig. S1 Box-plots showing all the physiochemical parameters of raw cow's milk evaluated in different dairy farm management throughout the year

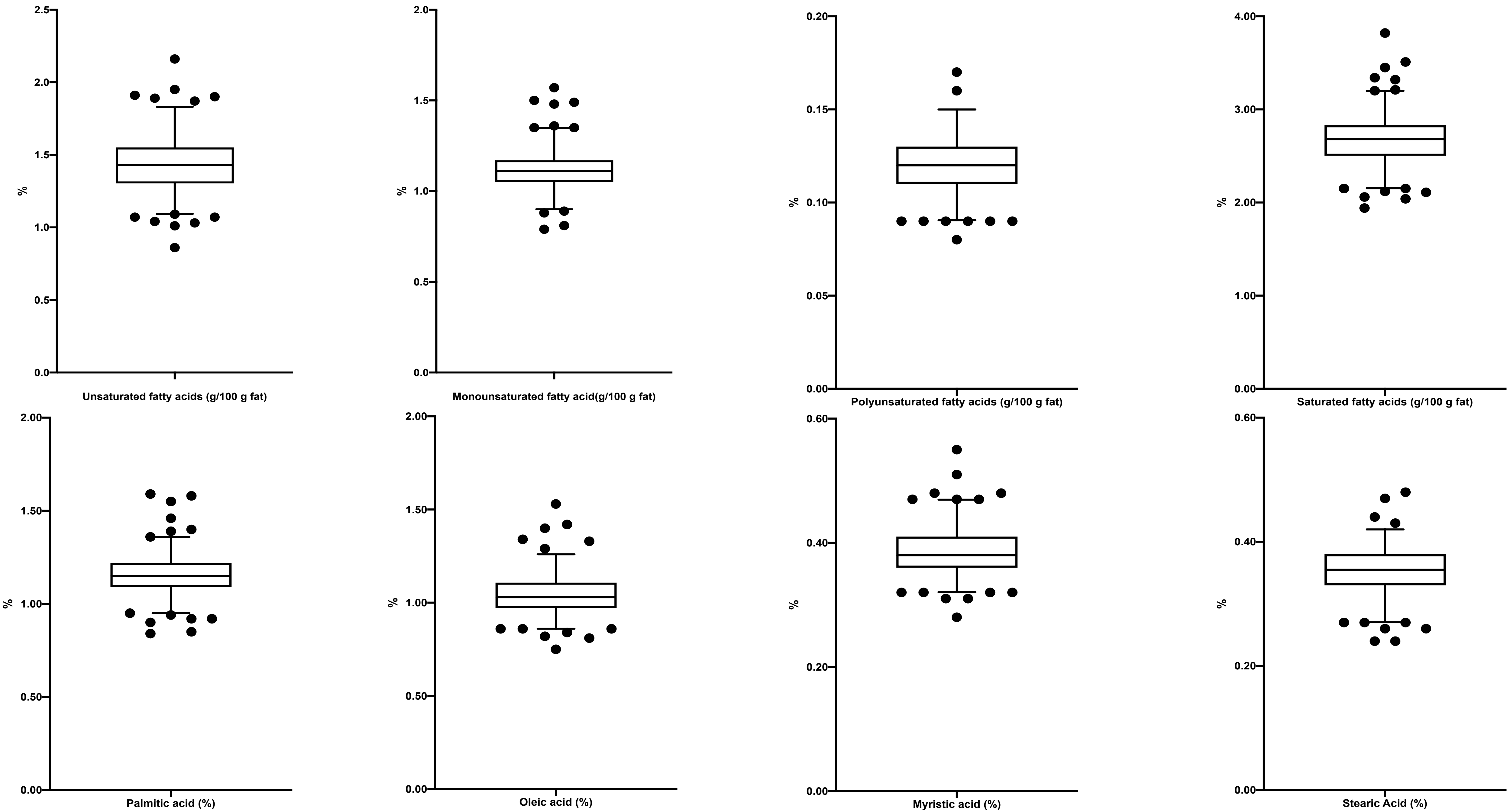

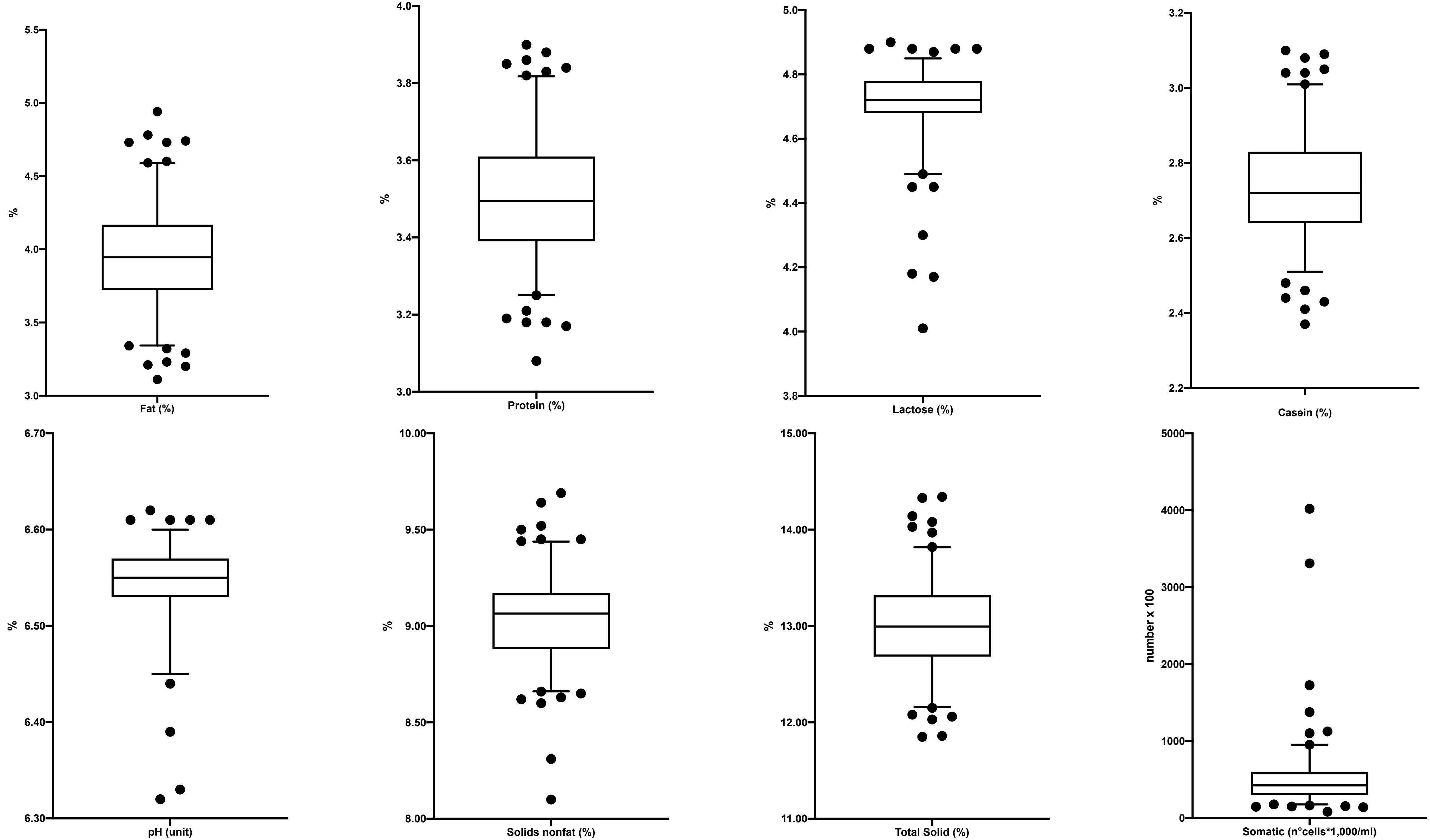

Fat (%)
Protein (%)
Lactose (%)
Casein (%)
pH (unit)
Solids nonfat (%)
Total Solid (%)
Somatic (n°cells*1,000/ml)
%
number x 100

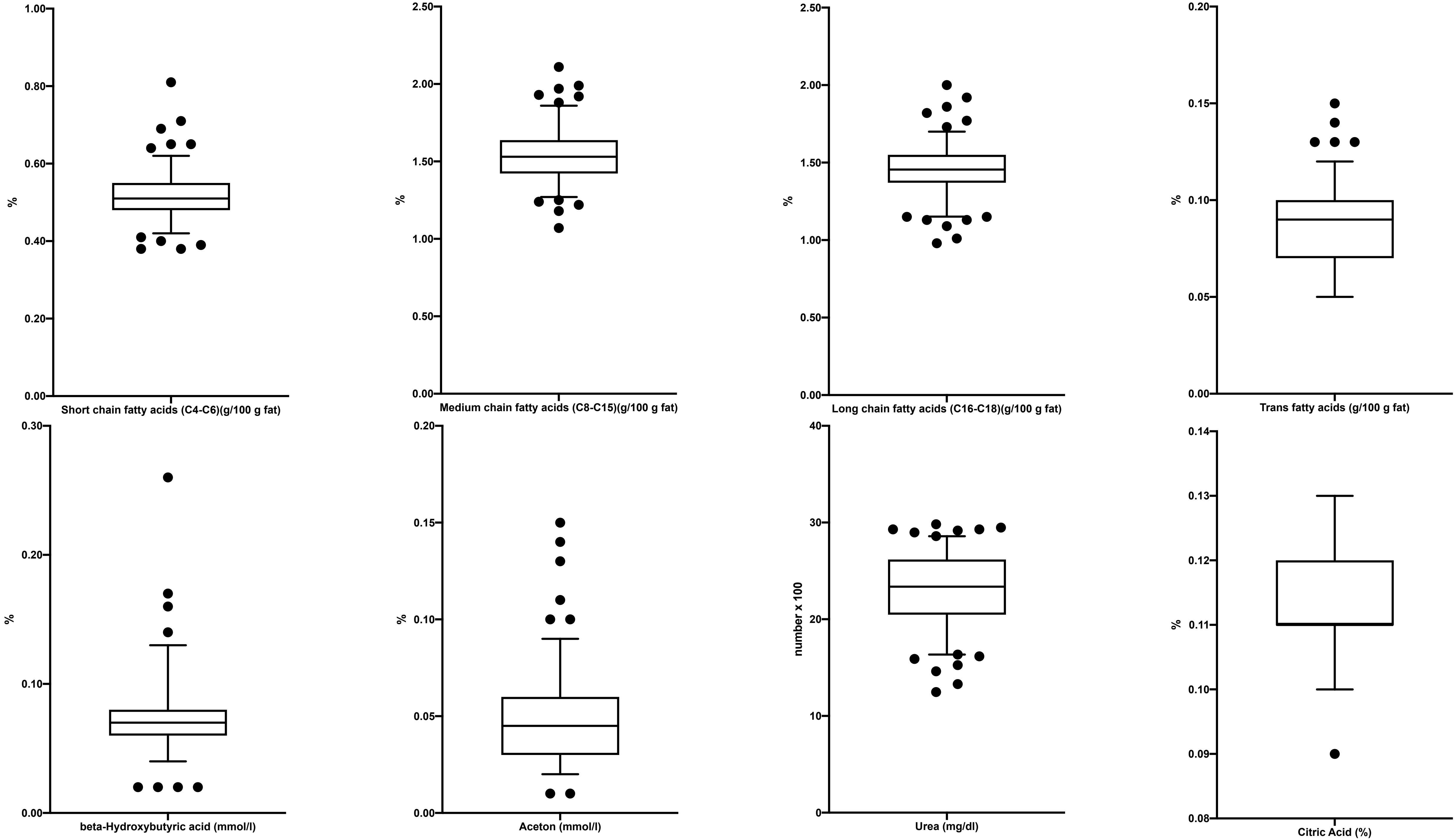
1.00
0.80
0.60
0.40
0.20
0.00
%
Short chain fatty acids (C4-C6)(g/100 g fat)
2.50
2.00
1.50
1.00
0.50
0.00
%
Medium chain fatty acids (C8-C15)(g/100 g fat)
2.50
2.00
1.50
1.00
0.50
0.00
%
Long chain fatty acids (C16-C18)(g/100 g fat)
0.20
0.15
0.10
0.05
0.00
%
Trans fatty acids (g/100 g fat)
0.30
0.20
0.10
0.00
%
beta-Hydroxybutyric acid (mmol/l)
0.20
0.15
0.10
0.05
0.00
%
Aceton (mmol/l)
40
30
20
10
0
number x 100
Urea (mg/dl)
0.14
0.13
0.12
0.11
0.10
0.09
0.08
%
Citric Acid (%)

FIG. S2

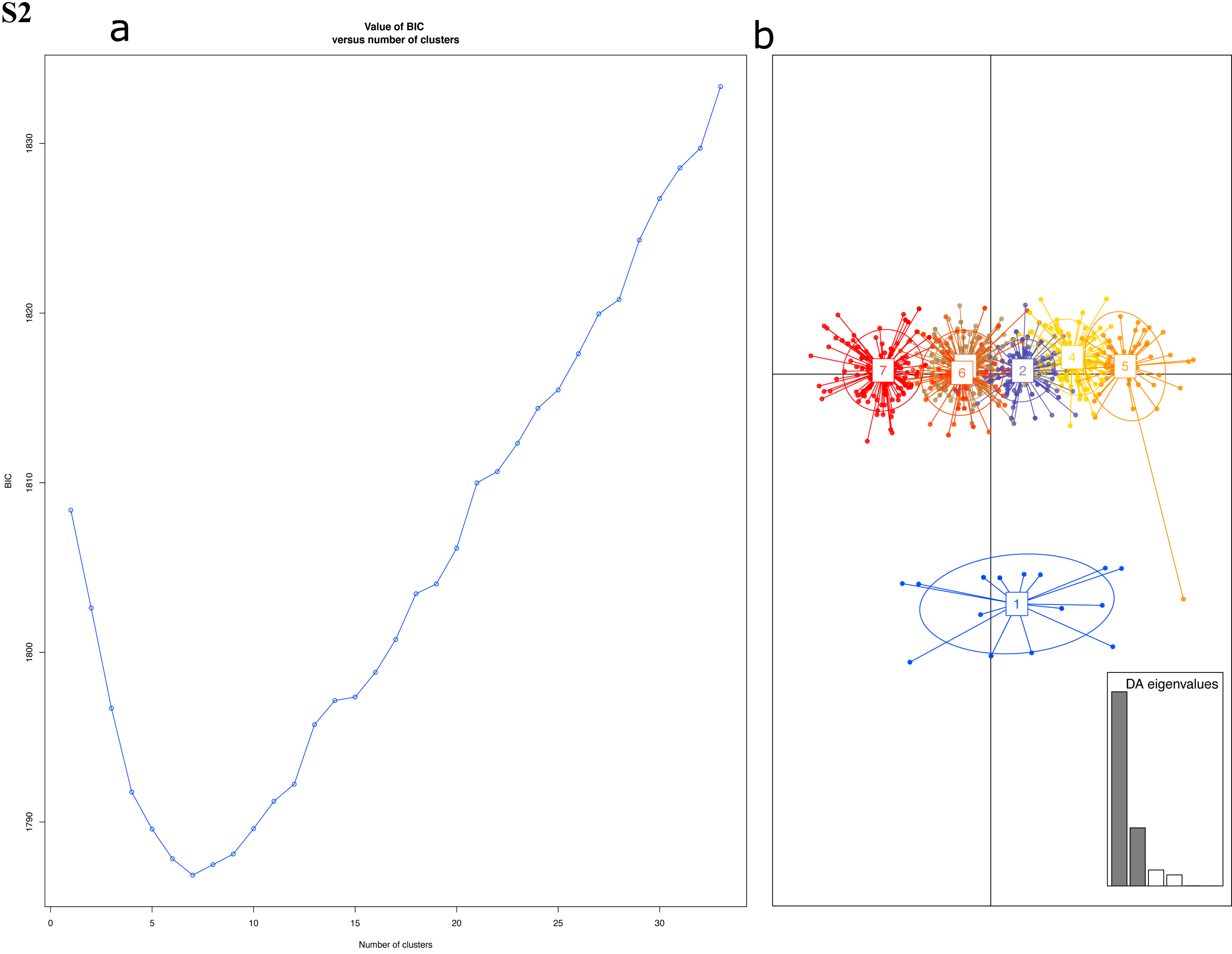

Supplementary figure 2. DAPC company clsuters resulted from BIC curve evaluation

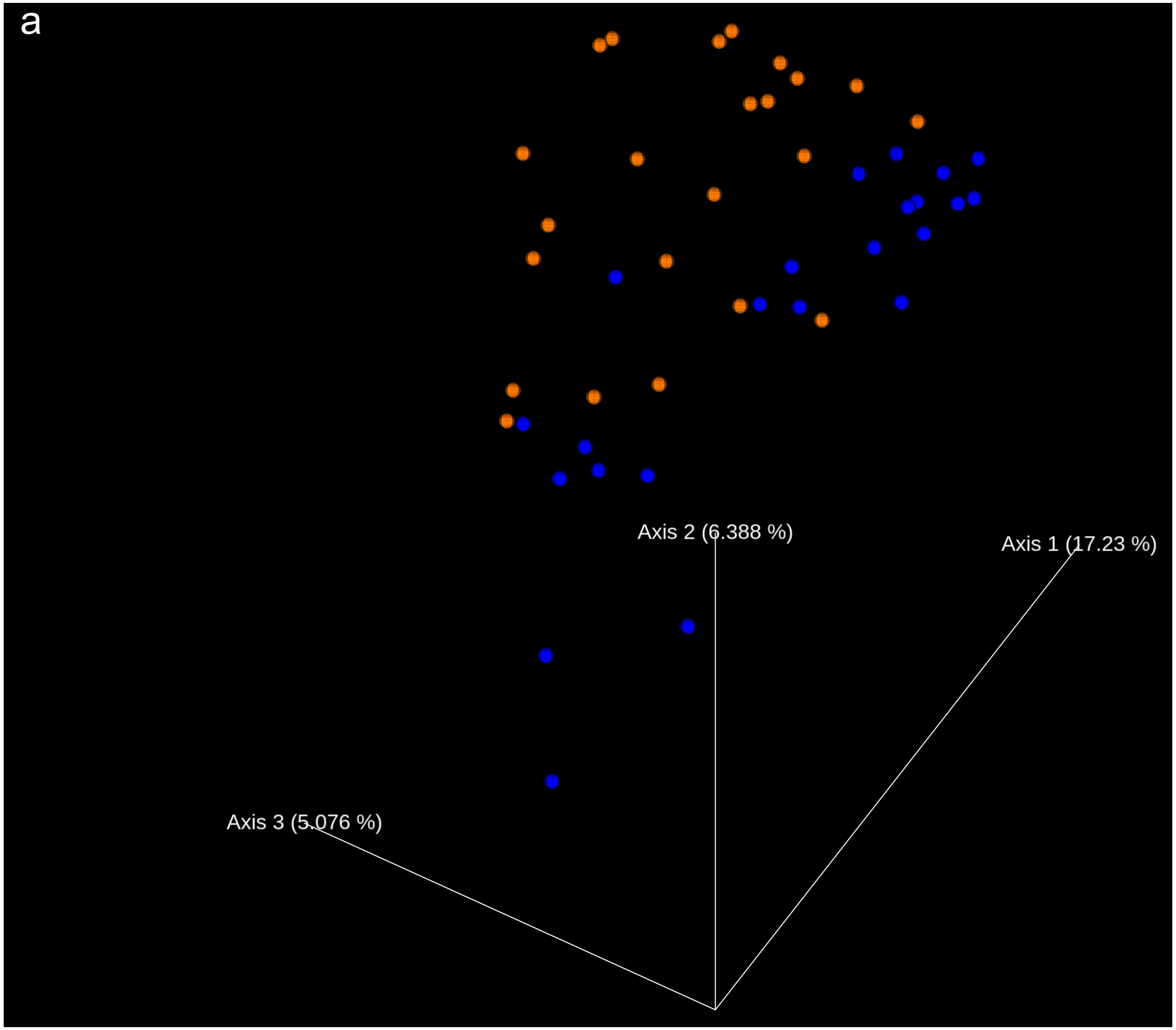

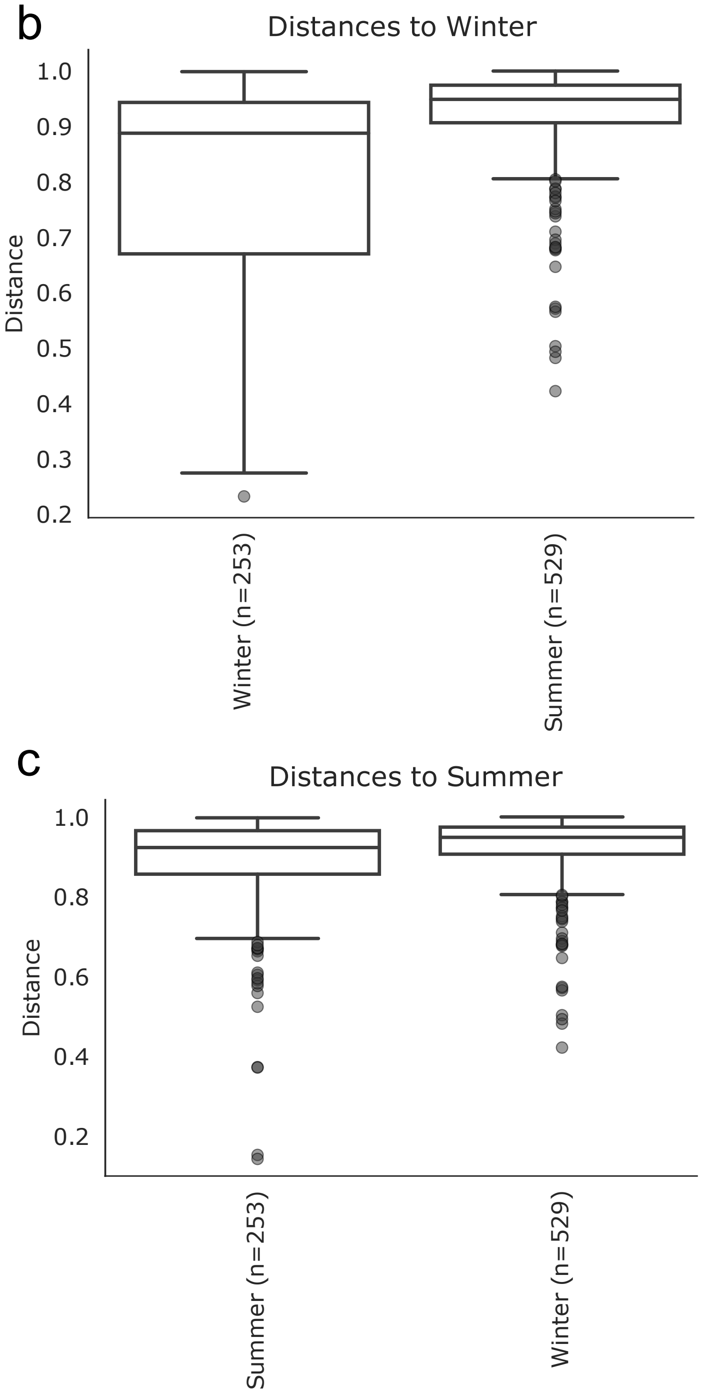

d

permanova-pairwise

Group 1	Group 2	Sample size	Permutations	pseudo-F	p-value	q-value
**Summer**	Winter	46	9999	5.222140541095830	0.0001	0.00015000000000000000

Supplementary figure 3. Emperor plot of beta diversity relative to winter vs. summer milk samples. PCA
based on the unweighted unifrac distance matrix describing winter (blue) vs. summer (orange) sample beta diversity is reported in panel a. Panel b and c describe the distance distribution obtained for the two seasonal groups with a PERMANOVA pairwise test whose statistical parameters are reported in panel d.

FIG. S4

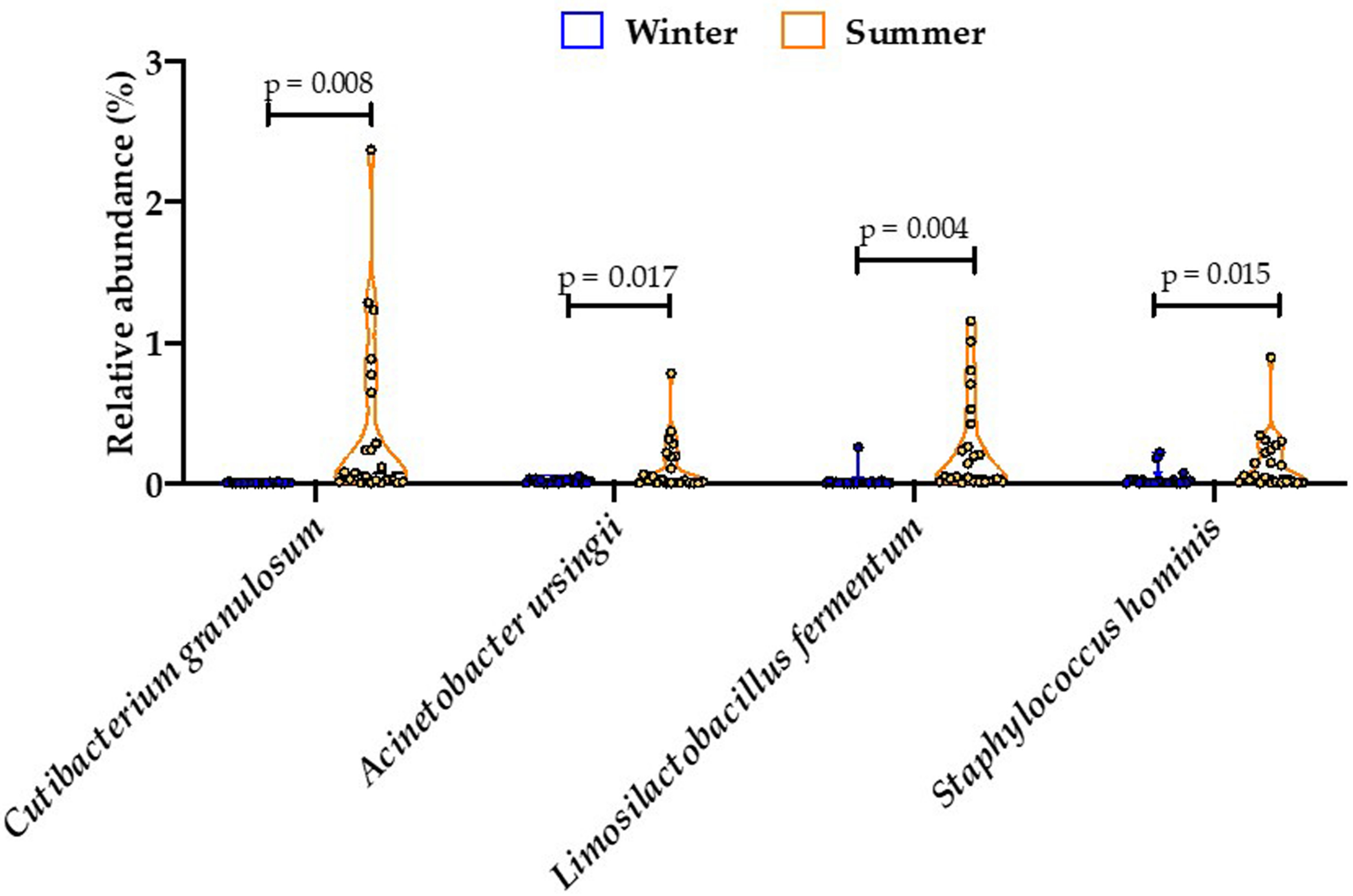

Supplementary figure 4. Relative abundance (%) of the OTUs assigned at species level that statistically ($p < 0.05$) differed between winter and summer raw cow milks samples
